# FruitPhenoBox – a device for rapid and automated fruit phenotyping of small sample sizes

**DOI:** 10.1186/s13007-024-01206-2

**Published:** 2024-05-23

**Authors:** Norbert Kirchgessner, Marius Hodel, Bruno Studer, Andrea Patocchi, Giovanni A. L. Broggini

**Affiliations:** 1 Crop Science, Institute of Agricultural Sciences, ETH Zurich, Universitaetstrasse 2, Zurich, 8092 Switzerland; 2 Fruit Breeding, Research Division Plant Breeding, Mueller-Thurgau-Strasse 29, Agroscope, Waedenswil, 8820 Switzerland; 3 Molecular Plant Breeding, Institute of Agricultural Sciences, ETH Zurich, Universitaetstrasse 2, Zurich, 8092 Switzerland; 4 ETH Zurich c/o Agroscope, Mueller-Thurgau-Strasse 29, Waedenswil, 8820 Switzerland

**Keywords:** *Malus domestica* Borkh., Imaging, Apple, Fruit shape, Fruit weight, Fruit color, Pomology, Breeding, Genetic resources

## Abstract

**Background:**

Fruit appearance of apple (*Malus domestica *Borkh.) is accession-specific and one of the main criteria for consumer choice. Consequently, fruit appearance is an important selection criterion in the breeding of new cultivars. It is also used for the description of older varieties or landraces. In commercial apple production, sorting devices are used to classify large numbers of fruit from a few cultivars. In contrast, the description of fruit from germplasm collections or breeding programs is based on only a few fruit from many accessions and is mostly performed visually by pomology experts. Such visual ratings are laborious, often difficult to compare and remain subjective.

**Results:**

Here we report on a morphometric device, the FruitPhenoBox, for automated fruit weighing and appearance description using computer-based analysis of five images per fruit. Recording of approximately 100 fruit from each of 15 apple cultivars using the FruitPhenoBox was rapid, with an average handling and recording time of less than eleven seconds per fruit. Comparison of fruit images from the 15 apple cultivars identified significant differences in shape index, fruit width, height and weight. Fruit shape was characteristic for each cultivar, while fruit color showed larger variation within sample sets. Assessing a subset of 20 randomly selected fruit per cultivar, fruit height, width and weight were described with a relative margin of error of 2.6%, 2.2%, and 6.2%, respectively, calculated from the mean value of all available fruit.

**Conclusions:**

The FruitPhenoBox allows for the rapid and consistent description of fruit appearance from individual apple accessions. By relating the relative margin of error for fruit width, height and weight description with different sample sizes, it was possible to determine an appropriate fruit sample size to efficiently and accurately describe the recorded traits. Therefore, the FruitPhenoBox is a useful tool for breeding and the description of apple germplasm collections.

**Supplementary Information:**

The online version contains supplementary material available at 10.1186/s13007-024-01206-2.

## Background

The appearance of apples (*Malus domestica* Borkh.) for fresh consumption is one of the first drivers of consumer choice in the supermarket [1]. Fruit appearance, defined as shape, color and size, is accession-specific [2] and an important trait when making selections in breeding programs [3, 4]. It is also an important trait for the description of older varieties or landraces [5–7]. In this context, single fruit are assessed visually to describe the color, while the size is measured using a ruler. Fruit shape is assessed by comparison with reference images. In commercial production, appearance is exploited for classifying fruit into different categories based on weight, caliber and color. Commercial sorting devices also allow for assessing defects and storage disorders [8], by analyzing several images of the same fruit. Such devices are designed for handling tons of fruit from a few cultivars and sorting parameters must be adjusted for each cultivar individually. However, in apple breeding programs and for the description of germplasm collections, requirements in terms of sample size are different: the appearance of a few fruit from many diverse cultivars, accessions or landraces, sometimes even from single trees, needs to be described. Commercial sorting devices, including belt or water conveyors, can be several tens of meter long, require automation for further processing/packing of the sorted fruit, and are very expensive. Smaller sorting devices lacking automation, as used for evaluating extension trials or for breeding purposes, require at least two to three persons to operate. Commercial sorting devices exploit proprietary software for fruit analyses which normally discard all raw data after generating a summary of the sorting results, and so they are not available for research or breeding purposes. Further, the space required as well as the acquisition costs of a sorting device can be prohibitive if purchased only for the description of a limited number of fruit per accession. Morphometry of fruit appearance on a limited number of fruit per category is mostly performed by analysis of images obtained either by placing sliced fruit on a scanner, as performed for the Tomato Analyzer [9, 10], or by imaging single fruit from different perspectives with a conventional SLR camera [11]. However, both are laborious methods and in the first case destructive. Therefore, the aim of this study was to develop a compact device, which enables automated, rapid and consistent phenotypic description of apple fruit. In particular, we aimed to (i) weigh individual fruit; (ii) image individual fruit from different angles; (iii) develop an automated image analysis software to extract fruit shape for each investigated cultivar; (iv) generate a factsheet summarizing the appearance of each accession; (v) assess performance of the FruitPhenoBox; and (vi) determine the minimum number of fruit per sample required for a confident fruit appearance description.

## Results

### Construction and operation of the FruitPhenoBox

The FruitPhenoBox was constructed following selecting, provisioning and combining commercially available components. The overall cost for the hardware required to build the FruitPhenoBox was below 5,000 USD. The software development in Matlab was done stepwise over several months, for a total time investment of two full weeks and eventually converted to a standalone version working in the Matlab runtime environment, so it can be freely used. In its final version, the space requirement for the FruitPhenoBox is less than a square meter and can be placed on a wheel-equipped table. Its operation requires a single person without specific informatics knowledge, and the use of a barcode to record the sample identifier (ID) avoids the need for manual typing during operation. The use of a foot pedal to trigger the image recording enabled the operator to use both hands to rapidly replace the fruit in the FruitPhenoBox.

### Performance assessment

**Fig. 1:**
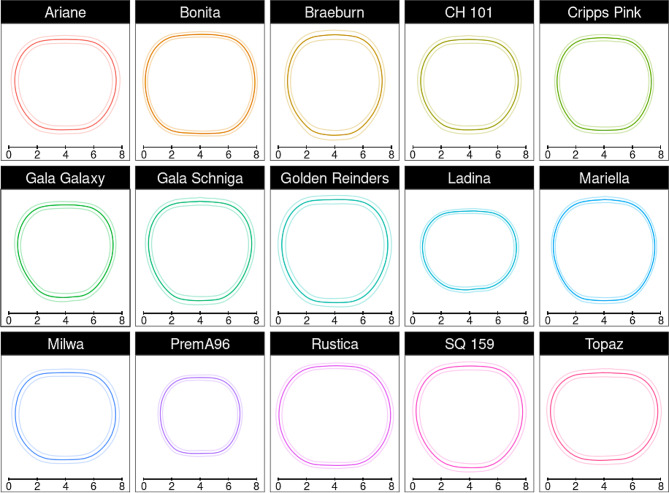
Fruit side shape of 15 apple cultivars (names given in the top bar). The average fruit side shape (solid line) and the angle-specific standard deviation (lighter lines) are based on the polar coordinates extracted from four side images per fruit and approximatively 100 fruit per cultivar. The bar beneath each average fruit shape indicates the scale in centimeters

**Fig. 2:**
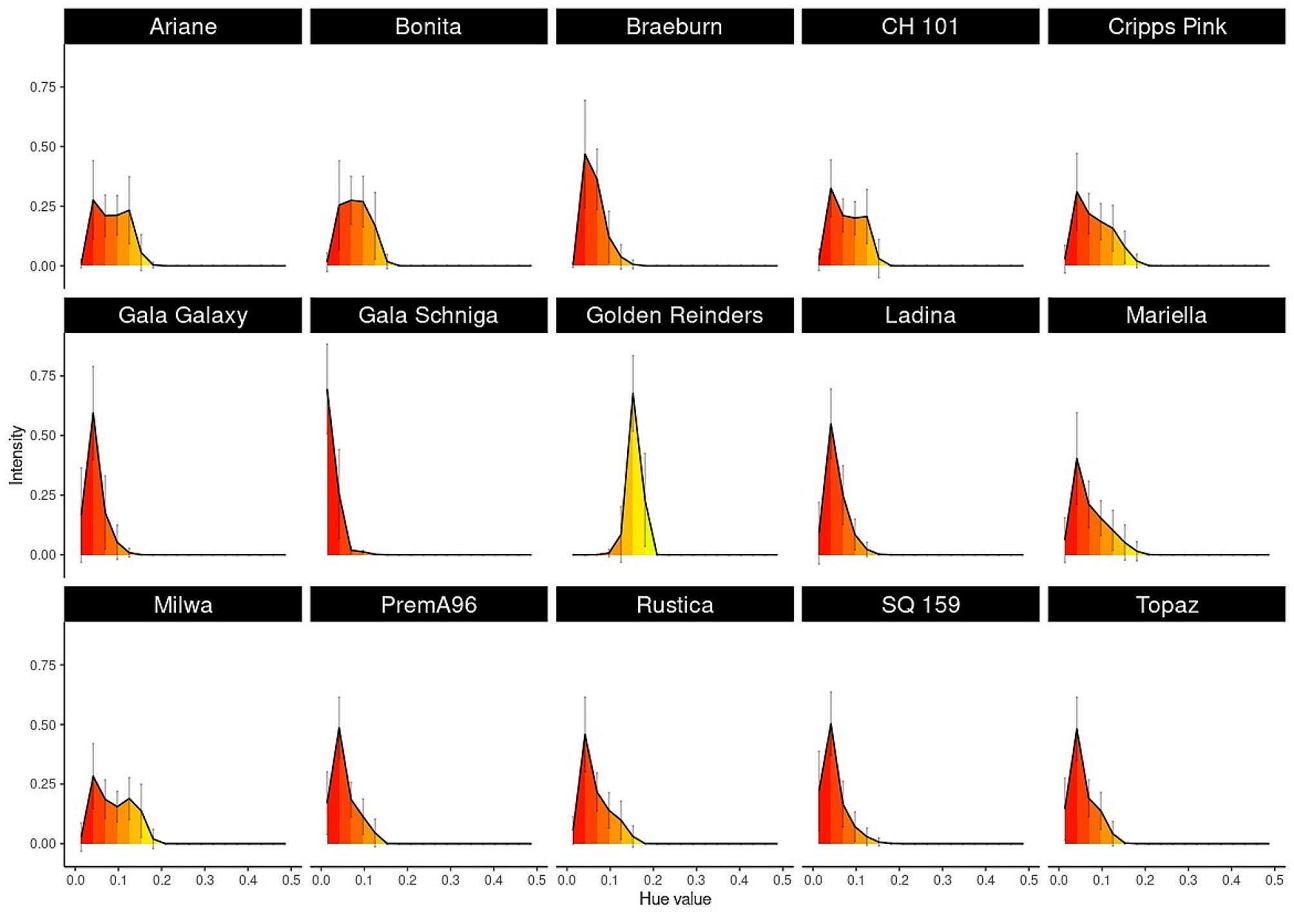
Histograms representing the hue value averages and standard deviations for 15 apple cultivars (names given in the top bar), calculated using four segmented fruit side images per fruit, on approximately 100 fruit per cultivar. The range of hue is normalized between 0–1 = (0-360°) and the hue value interval between 0.5-1 (cyan, blue magenta) is not shown, as yellow, red and green are the most relevant apple peel colors

Images and weights of approximatively 1,500 apple fruit from 15 cultivars were recorded over two sessions by a single operator with an average elapsed time of 10.58 s (median 9 s) per recorded fruit (replacing the fruit in the FruitPhenoBox, followed by image and weight recording). A distinctive average fruit shape (including angle-specific standard deviations) was generated for the fruit of each cultivar from the side view images (Fig. 1). The comparison of the average fruit shapes of all cultivars to 13 reference shapes (Figure S1) revealed that the most represented shapes belonged to the classes “rectangular”, “rectangular conical” and “flattened spherical”, with four cultivars assigned to each class (Factsheet, Figure S2). The average shapes of all fruit were then used to perform a k-clustering analyses, generating between three and eight clusters (Figure S3). Using six clusters, the number of fruit of a cultivar assigned to each cluster was counted (Figure S4). One-way ANOVAs on fruit asymmetry revealed significant differences between cultivars, and Post hoc Tukey test generated up to seven significance groups (*p* < 0.05), with median asymmetry values ranging between 0.016 and 0.022 (Figure S5). One-way ANOVAs on height, width, weight and shape index revealed that there are significant (*p* < 2e-16) differences between cultivars for all these traits. Post hoc Tukey test generated up to ten significance groups (*p* < 0.05) per investigated trait (Figure S6). The average color distribution of the hue values per cultivar showed large deviations among but also within cultivars (Fig. 2).

### Determination of the optimal sample size

**Fig. 3:**
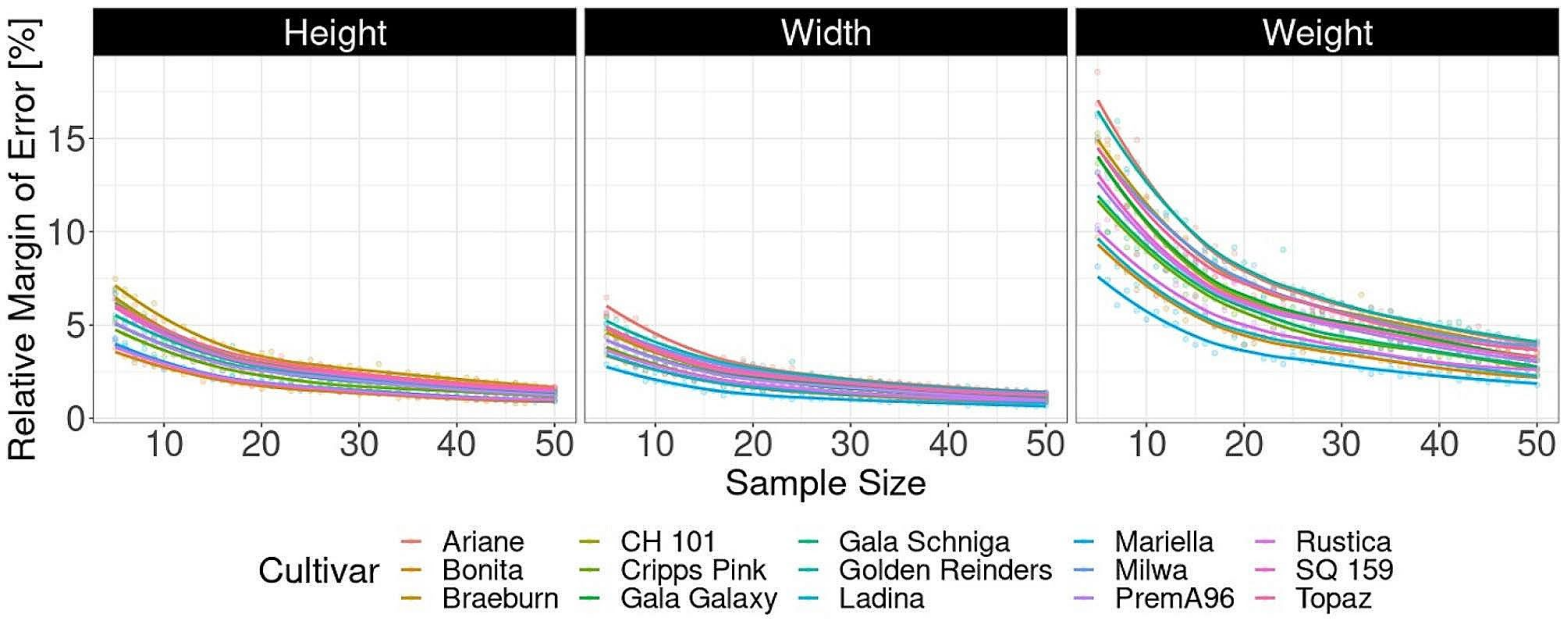
Effect of sample size on relative margin of error for fruit weight, width and height across the 15 investigated cultivars based on 200 random samplings per sample size and cultivar. The relative margin of error is the radius of the 95% confidence interval for each sample size divided by the corresponding mean values calculated using all available (approximatively 100) fruit of each cultivar

Testing the effect of different sample size of the 15 apple cultivars on the relative margin of error of the fruit description indicated a general improvement with increasing sample sizes, particularly between n = 5 and n = 20 (Fig. 3). The average fruit height, width, and weight calculated from 20 randomly selected fruit was described with a relative margin of error of 2.6%, 2.2%, and 6.2%, respectively, of the mean values calculated using all available fruit of a cultivar. The largest margins of error were observed for fruit height of the cultivar ‘Braeburn’ (3.5%), and for both fruit width and weight of the cultivar ‘Golden Reinders’ (2.9% and 8.3%, respectively). The cultivars showing the lowest margins of error were ‘Bonita’ for fruit height (1.6%), and ‘Mariella’ for fruit width and weight (1.4% and 3.9%, respectively).

## Discussion

The FruitPhenoBox is a compact device enabling rapid and consistent description of apple fruit appearance using a system based on a combination of Matlab and R scripts. Compared to existing commercial sorting devices, this system is less expensive, more compact, operable by a single user and can be implemented for the semi-automated description of fruit from a relatively small number of fruit per sample from a large number of cultivars, accessions or landraces.

The use of a barcode-based labeling of the fruit samples reduces naming mistakes. A total of approximatively 1,500 fruit can be processed by a single operator within a few hours, achieving a fast and objective appearance description based on features (as fruit dimensions and weight, fruit coloration and side shape) extracted from the recorded images. The FruitPhenoBox offers the important feature of storing the fruit images as high-quality image files, ensuring the possibility of documenting the description process, and to allow a later re-analysis in case additional information needs to be extracted using novel algorithms, methods, or novel reference shape descriptors. The images generated could be used for further applications, e.g., training machine learning algorithms for the development of an automated cultivar recognition algorithm based on fruit features. Alternatively, extracted fruit appearance features could be combined with genotypic information for a genome-wide association study. The FruitPhenoBox was already applied to extract fruit shape and colors features from a subset of the Apple REFPOP [12]. The combination of these features with the available genotypic information in a GWAS allowed unraveling the genetic architecture of shape and fruit color [13] appearance traits.

Our analysis performed on approximately 100 fruit from 15 apple cultivars each confirmed that fruit shape is characteristic for each cultivar [2]. For single recordings, the fruit segmentation and the shape description resulting thereof showed disrupted patterns (e.g, cyan blue and orange top contours for the cultivars SQ159 and Topaz, respectively, Factsheet, Figure S2). The rare occurrence of such issues should, however, not impact the final outcome and could be solved by adjusting the segmentation procedure. The comparisons of the maximum correlation of side views to 13 shapes descriptors (Figure S1) or of six side fruit shapes resulting from k-mean clustering (Figure S 4) revealed a marginal improvement using the latter (Figure S7). This confirms that the 13 shapes descriptors are valuable references and should be used to ensure continuity with the previous descriptions. The color distribution showed large variation within cultivars, confirming the common knowledge that despite being cultivar-characteristic, fruit coloration can be affected by external factors such as exposure to sun and, therefore, shows within-tree variability [14–16]. Generating a histogram for each fruit allowed depicting intra-fruit color variability, and as expected, ‘Gala Schniga’, a deep red mutant of ‘Gala’, generated a histogram with a narrower peak around the red hue value compared to ‘Gala Galaxy’. The recorded images could be analyzed separately for other purposes, e.g., the dissection of fruit coloration in the three main colors (green, yellow and red), as commonly used in sorting devices, or the distinction of ground color from overcolor.

Fruit asymmetry showed large variation within the fruit of a single accession, reflecting the common knowledge that fruit asymmetry is resulting from the fruit deformation during the development as consequence of physical contact to branches or other structures close to the tree.

Furthermore, our investigation showed that the fruit sample size had stronger influence on the fruit weight description confidence compared to fruit width or height. This is because weight is correlated to volume, which varies cubically with changes in width or height. On this basis, we propose a minimal sample size of 20 fruit per sample for consistent fruit description. In general, this number of fruit can be harvested from a single tree. The factsheet, summarizing in one page the data from each cultivar, offers a swift way to compile a basic overview of essential fruit characteristics when describing for instance apple genetic resources.

The FruitPhenoBox was also used to record images from pears (*Pyrus communis*), requiring only the adaptation of the downstream analysis for fruit feature extraction, whose description go beyond the scope of this manuscript. We evince that with similar adaptations, the scripts required for image analysis could be rapidly adjusted to extract shape features from images recorded from other fruits or vegetables.

## Conclusions

The FruitPhenoBox is a morphometric device combining low hardware acquisition costs, compact design, and single-user and rapid operation. It enables an automated analysis avoiding any user bias and generates consistent data for describing accession-specific fruit appearance from fruit sample sizes of approximately 20 fruit. Therefore, the FruitPhenoBox is a useful tool for breeding and the description of apple germplasm collections.

## Materials and methods

### Plant material

About 100 fruit for each of the 15 apple cultivars ‘Ariane’, ‘Bonita’, ‘Braeburn’, ‘Milwa’ (Diwa®), ‘Gala Galaxy’, ‘Gala SchniCo Schniga’, ‘CH 101’ (Galiwa®), ‘Golden Reinders’, ‘Ladina’, ‘Mariella’, ‘SQ159’ (Natyra®), ‘Cripp’s Pink’ (Pink Lady®), ‘PremA96’ (Rockit®), ‘Rustica’ and ‘Topaz’ were produced in 2021 according integrated production practices in orchards of Agroscope, Wädenswil, Switzerland.

### Hardware description

**Fig. 4:**
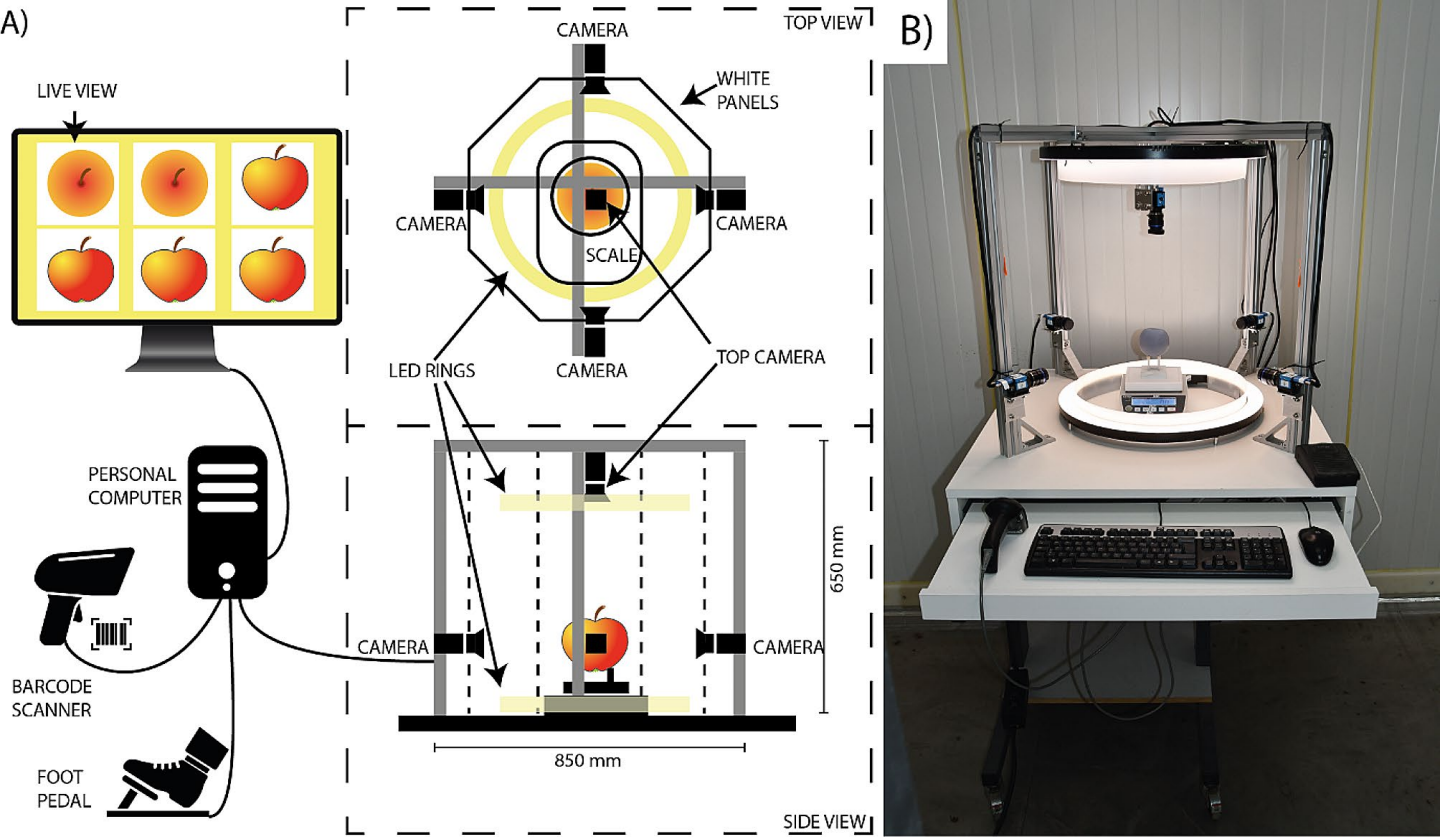
(**A**) Schematic representation of the FruitPhenoBox consisting of five cameras mounted on an aluminum frame. The FruitPhenoBox allows imaging single fruit placed on a pedestal located on the scale in the center of the frame from five different sides. Two LED rings ensure homogeneous illumination of the fruit. The cameras and scale are connected to a personal computer. The sample unique identifier is recorded using a barcode scanner, while imaging and weight acquisition is triggered by a foot pedal. (**B**) Image of the inner structure of the FruitPhenoBox (without the white PVC panels)

The FruitPhenoBox (Fig. 4) consists of an external frame of 850 × 850 × 660 mm built of aluminum profiles (30 × 30 mm) mounted on a wooden base plate. Five rgb cameras, one from each side and one from the top (DFK 33UX273, 1296 × 1080 pixel + TCL 0814, www.theimagingsource.com) are mounted on the aluminum profiles and oriented toward the center, where a weight scale with serial connection port (Kern PCB 1000-1, www.kern-sohn.com) and a pedestal is placed. Cameras and scale are connected to a common personal computer. Two LED-rings (YONGNUO YN308, www.yongnuo.eu) provide homogeneous lighting from the bottom and from the top. A barcode scanner (QuickScan Lite QW2400, www.datalogic.com) is used for registering unique ID/Cultivar name/accession number/QR code and a USB pedal (6210–0084 USB Footswitch, www.herga.com) is used as trigger. White PVC panels (3 mm) with camera holes (39 mm diameter fitting camera lens) shield the FruitPhenoBox from external light disturbance. For stability, the device was mounted on a piece of furniture, allowing storing the personal computer and a pull-out keyboard tray.

### Image acquisition and analysis

Image acquisition and analysis are steered by two different Matlab [17] scripts that were compiled to run in the Matlab Compiler Runtime 2018b environment. The first script controls the hardware as follows: Prior photograph shooting, camera parameters are defined to fix white balance parameters at first run and reload them afterwards. Prior to the measurement process, a unique barcode (ID) assigned to each single tree name or cultivar is scanned with a 2D-scanner. A fruit is placed on a pedestal on the scale in the center of the FruitPhenoBox, and the foot pedal triggers the snapshot from the five cameras as well as the recording of the fruit weight. This latter is recorded in the image metadata. Image names are generated concatenating ID, camera number, an incremental number starting from one, the shooting date and time, and are stored in 48-bit TIF format in a folder “images” and distributed in subfolders according to the barcode and the camera used for shooting (e.g., images/ID/cam1). Each snapshot generated five lossless compressed (packbits) TIF-images (1296 × 1080, 48-bit, each sizing approximatively 8 Mbytes each), four from each side and one from the top. Once all fruit are imaged, each single image is then segmented using a second Matlab script (https://sourceforge.net/projects/affe) to remove the image background, define fruit position and outline its contours. Segmentation is done using hsv-color space in several steps: the ratio of saturation and value was thresholded with 0.2 and intersected with all values of saturation above 0.19 resulting in the first mask m_1. Additionally for each camera an image of the empty FruitPhenoBox (taken without apple) is subtracted from the actual image and the difference segmented with Otsu’s method [18] resulting in a second mask m_2. The set union of both masks was calculated as apple area in each image. Distribution of pixels color of the fruit area was analyzed and density of the hue values was used to generate a histogram per each single fruit combining the information from the four fruit side views. The range of hue was normalized between 0 and 1 = (0-360°) and only the hue value interval between 0 and 0.5 (yellow, red and green) was used to generate a histogram.

Width and height were calculated from each single image utilizing the real-world pixel size of calibration images.

Fruit side contours were converted from cartesian to polar coordinates (around their centroid) and interpolated to integer degree values for simple further analysis. For side views the contour was corrected to minimize asymmetry to the y-axis by rotation around its centroid, the fruit mask was subsequently rotated by the determined angle. As an indicator of fruit asymmetry, the fruit masks of side views were mirrored vertically at the axis parallel to the y-axis through their centroid. The area of the differences of original mask and mirrored mask was divided by the double area of the original mask yielding 0 for perfect symmetric shapes and 1 as maximum for this indicator for very unsymmetric fruit shapes. The radius of the contours was compared to the shapes provided in pomological description manuals [7] and Figure S1, assigning fruit shape to one of the 13 available categories based on the highest correlation.

Fruit top contours coordinates were transformed into polar coordinates with origin in the center of mass of the apple area. Polar coordinates were used for determination of circumcircle (the smallest circle completely fitting outside the fruit top contours), mean radius and incircle (the largest circle fitting completely inside the fruit top contours) as well as the deviations of the radius to the mean circle. The standard deviation of these deviations was calculated and normalized to radius 1. Then the single image analyses were grouped per fruit, and then a text file containing the information of the variables listed in Table 1 per fruit of each single cultivar was generated.

**Table 1:** Apple fruit output variables of the second Matlab script assessed with the FruitPhenoBox.

Variable name	Description
ShapeClassCorr_side	normalized cross-correlation coefficient of side shape with reference shapes
ShapeClass_top	id of best-fitting reference shape, top view
weight_g	mass of apple [g]
radius_mean_pix	mean radius top view [pixel]
radius_in_pix	incircle radius top view [pixel]
radius_circ_pix	circumcircle radius top view [pixel]
radius_std_nor_pix	standard deviation of radius of boundary in polar coordinates top view normalized to radius 1 [pixel]
width_cm	apple width of all side views [cm]
width_mean_cm	mean apple width [cm]
height cm	apple height of all side views [cm]
height_mean_cm	mean apple height [cm]
asymmetry	asymmetry value: difference area with mirrored mask / 2 / mask area
symmetry_angle_deg	rotation angle of mask to achieve the minimal asymmetry value
colhistheader	column names of color histograms
colhist	color histogram of hue and saturation of all views

### R-Script for results visualization

The output files of the apple fruit feature extractor Matlab script provide the input for an R script [19] to summarize and visualize the data. The script creates summary factsheets (Supplementary data) per tree or cultivar depending on user input. For this purpose, a text file with the field plan of the experimental site including tree name/ID, cultivar name, row and tree position is required as an additional input file (Table S1). The resulting factsheet contains information about fruit weight, width, height. Each top shape, an average side shape per fruit and an average side shape per single tree or cultivar (in case several trees of the same cultivar are investigated) is displayed. The average side shapes are determined using the polar coordinates; for this purpose, the average radius is calculated for each degree. Furthermore, the factsheet shows five photos, one from each side, of the first fruit per tree or cultivar placed in the FruitPhenoBox, a color histogram of the mean values and additional information about the shape and data collection. Overall, the factsheets give an overview of the data and a first impression of the accession-specific properties. Additionally, the R script exports polar coordinates, Cartesian coordinates, raw data of color histograms, correlation to predefined shapes and fruit weight and caliber in different text files. Thus, all the data collected is available in a compact and machine-friendly format for further analysis. The R script requires the packages dplyr [20] and tidyr [21] for data wrangling. The packages ggplot2 [22], gridExtra [23], gtable [24], qrcode [25], magick [26] and patchwork [27] are required for image processing, data plotting and creating the fact sheet.

### Performance assessment

To assess the performance of the FruitPhenoBox, approximatively 100 fruit of the 15 apple cultivars were photographed by a single user. More precisely, 91 and 97 fruit were available for ‘Bonita’ and ‘Golden Reinders’, respectively, while for all other cultivars 100 fruit were used. Time stamps were used to estimate the average time elapsed between the snapshot of two different fruit, including the time required for replacing the fruit in the chamber. Average shapes per cultivar were generated, together with a boxplot representation of the distribution of weight, width, height, and shape index (height/weight). One-way ANOVAs were performed in R [19] to analyze differences of the four parameters between cultivars. For each parameter, letters indicating significance groups (p < 0.05) were assigned according to a post hoc Tukey test.

### Optimal sample size determination

To determine the optimal sample size required to obtain confident results, we tested sample sizes (n) between five and 50 apples per cultivar. For each sample size, a random subset of n apples per cultivar was randomly selected from approximately 100 apples and the mean of their weight, width and height was calculated (200 times for each sample size). From the resulting mean sample values, the 2.5% and 97.5% quantiles and the means were calculated for each trait and cultivar. Description 95% confidence interval is the range between the lower and upper quantile divided by the mean value per cultivar and its half corresponds to the relative margin of error.

### Electronic supplementary material

Below is the link to the electronic supplementary material.

Supplementary Material 1

Supplementary Material 2

## Data Availability

Supplementary files for this article, which include raw images in tif format, datasets extracted from the images and R scripts used in this study are available from the ETH Research collection under following doi: 10.3929/ethz-b-000590509. The Matlab- and R-scripts are available via sourceforge, project apple fruit feature extractor (affe), https://sourceforge.net/projects/affe.
