# Supplementary material for: FruitPhenoBox – a device for rapid and automated fruit phenotyping of small sample sizes
Source: Plant Methods. 2024 May 23;20:74. doi: 10.1186/s13007-024-01206-2 (PMC11112871; doi:10.1186/s13007-024-01206-2)
Supplement: Supplementary file 1 — Supplementary Material 1 [file 13007_2024_1206_MOESM1_ESM.pdf]

Ariane

Row: 1
Tree: 1

Nr Apples:	Top Shape Class:	Symmetry:	Shape category:
100	3	normal	broad spherical−cone−shaped

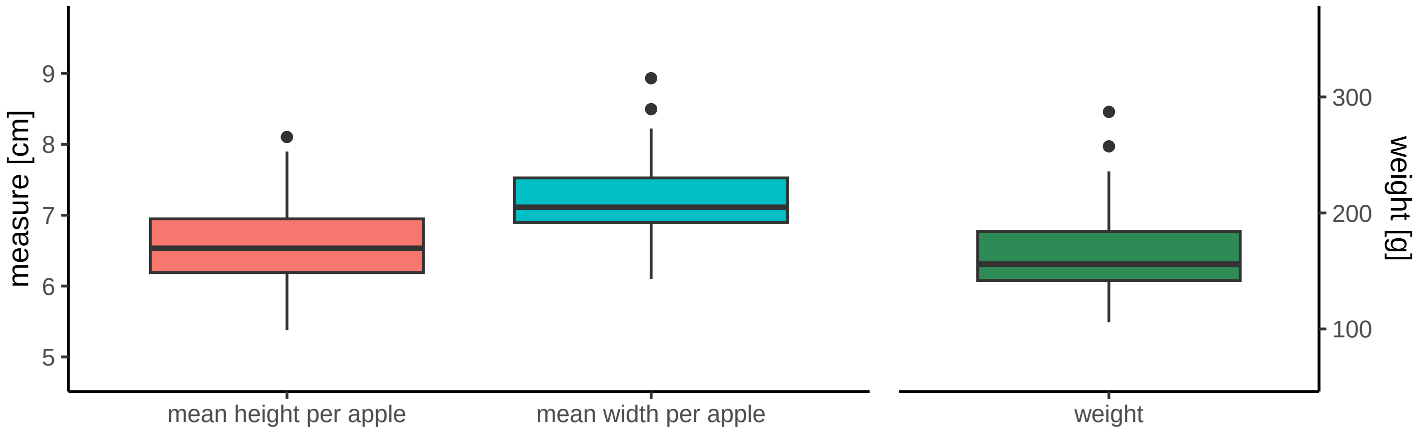

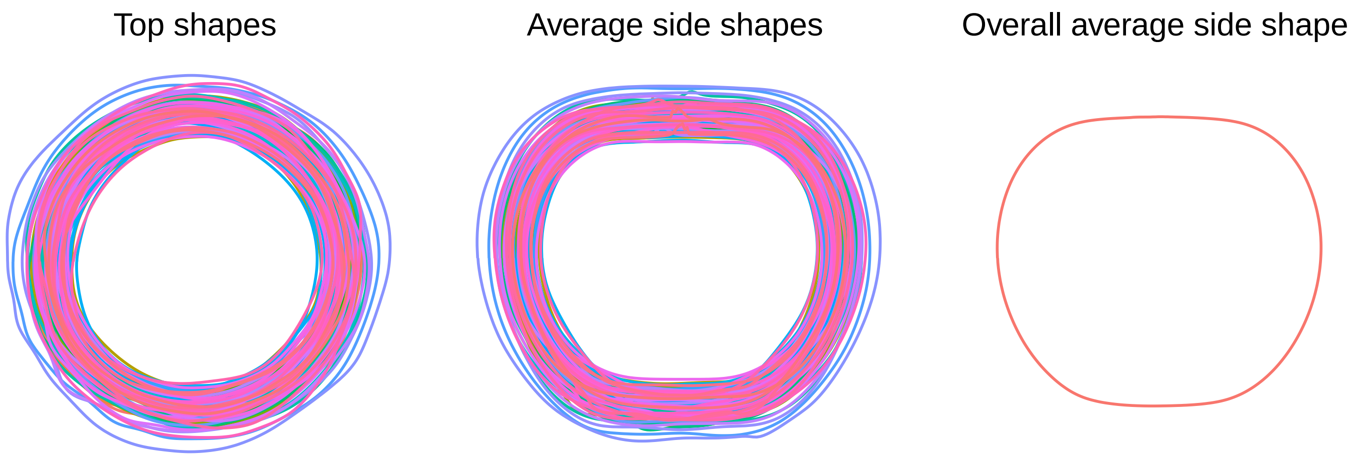

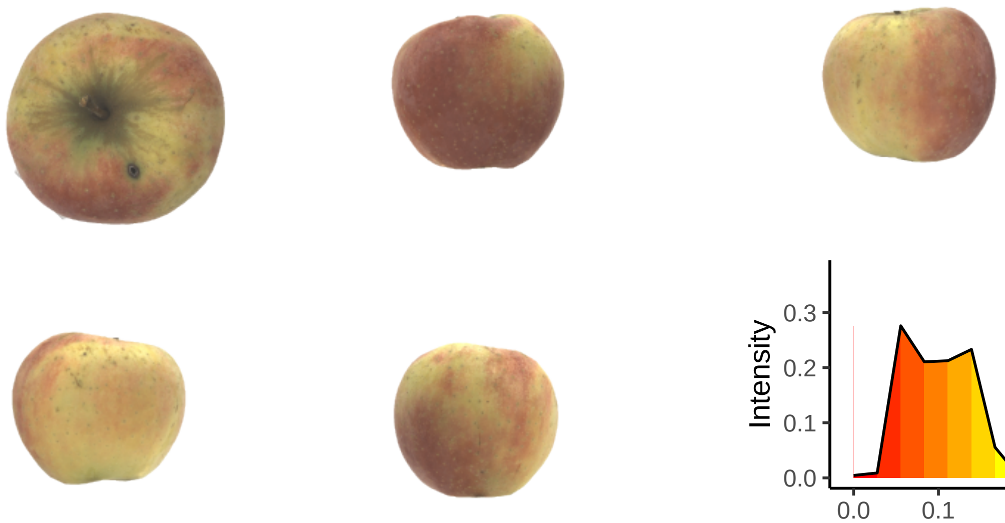

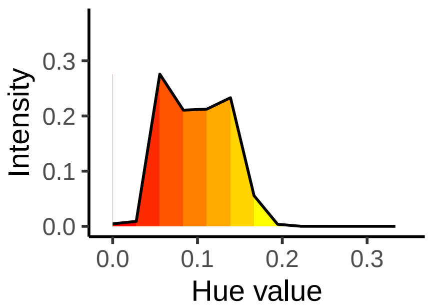

Location: Wädenswil
Year: 2021

Photos taken on 2021−01−06
between 15:06:57 and 15:25:59

Bonita

Row: 2
Tree: 2

Nr Apples:	Top Shape Class:	Symmetry:	Shape category:
91	3	normal	rectangular

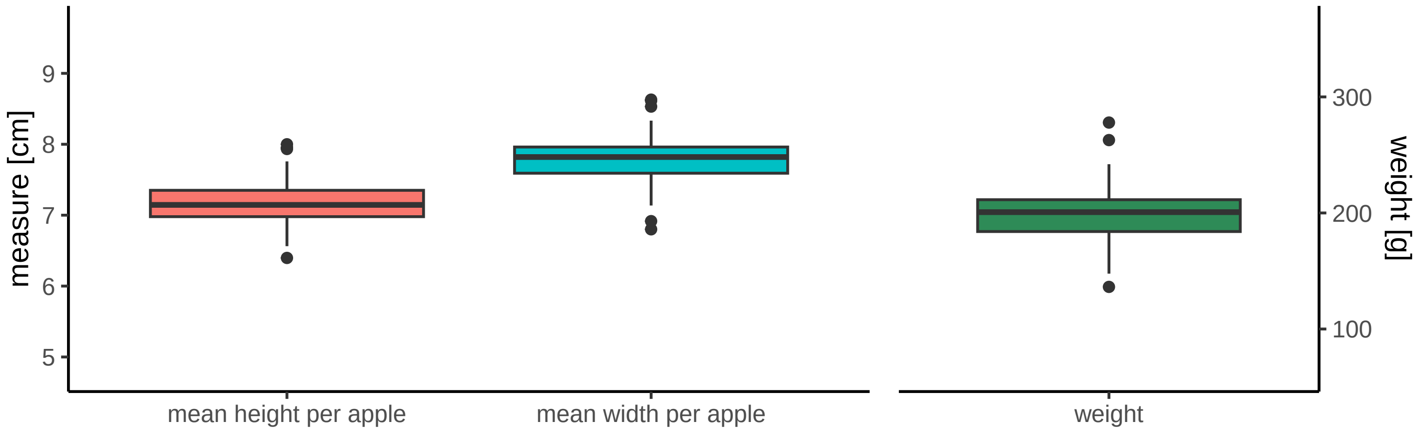

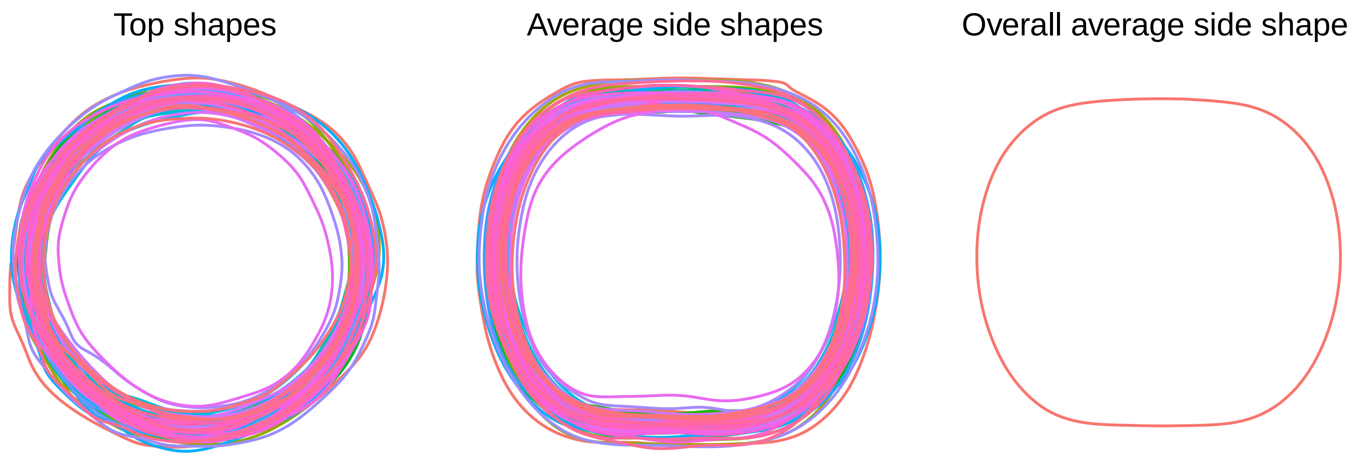

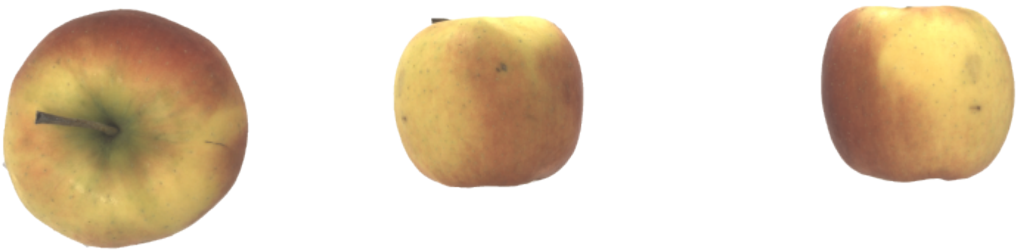

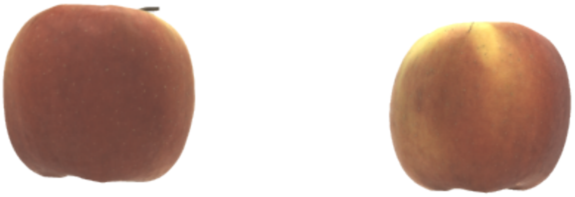

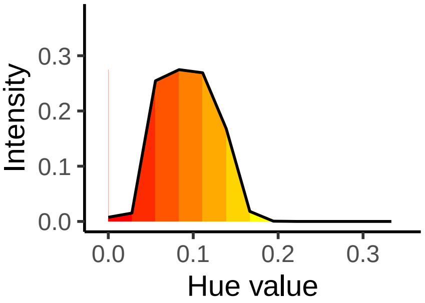

Location: Wädenswil
Year: 2021

Photos taken on 2021−01−06
between 16:02:30 and 16:19:42

Braeburn

Row: 3
Tree: 3

**Nr Apples:**	**Top Shape Class:**	**Symmetry:**	**Shape category:**
100	3	symmetric	obtuse conical

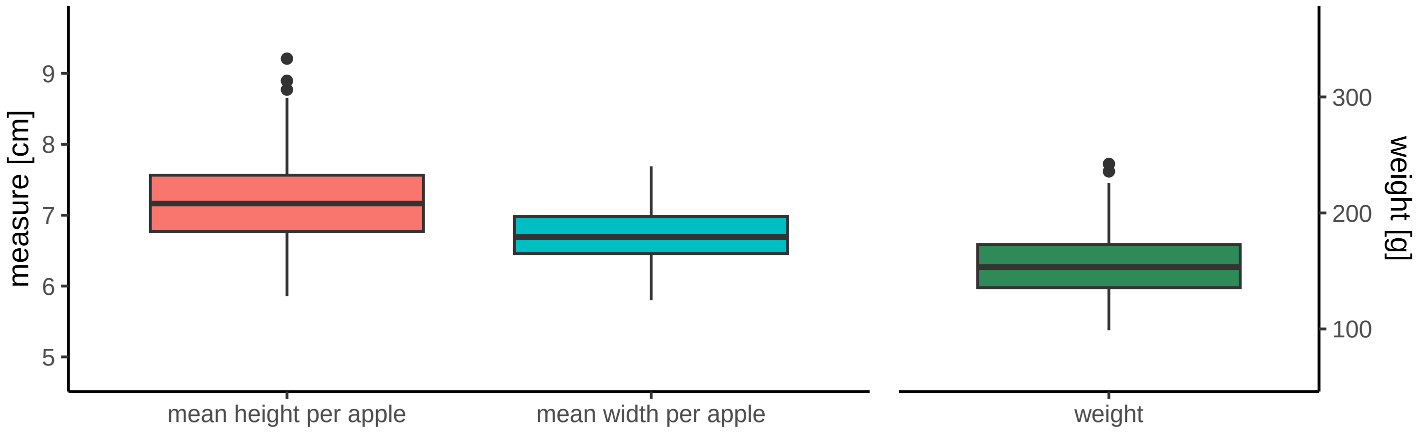

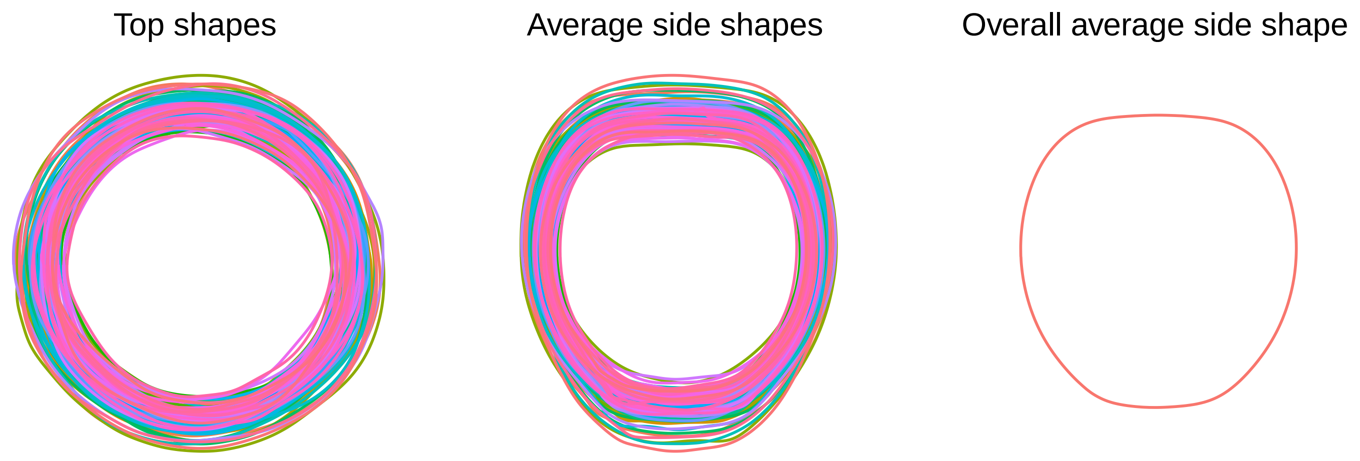

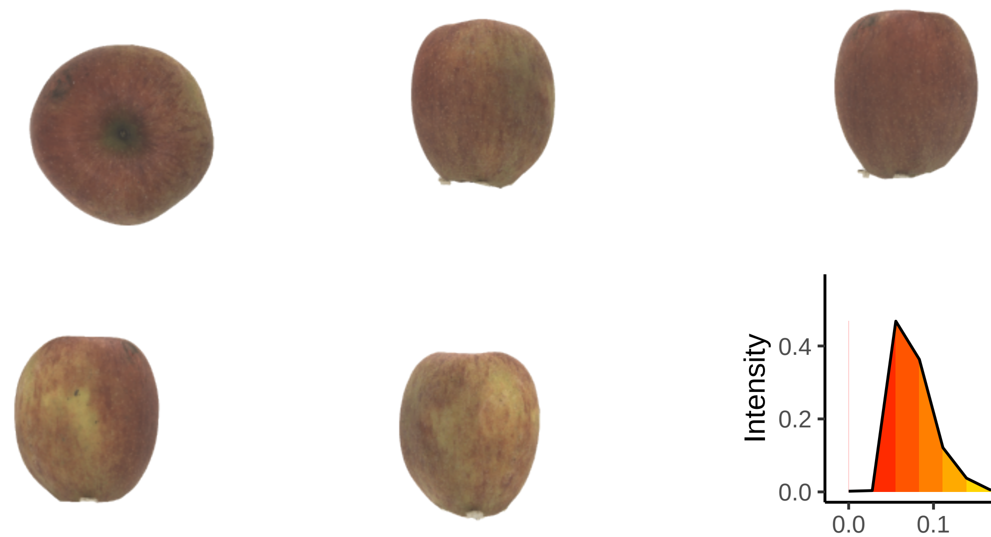

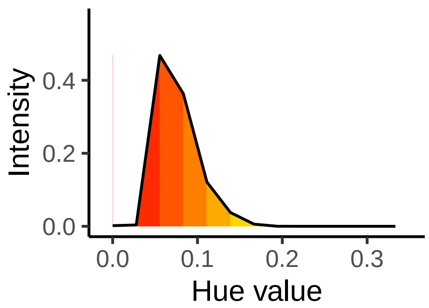

Location: Wädenswil
Year: 2021

Photos taken on 2021−01−06
between 14:50:21 and 15:06:08

CH 101

Row: 4
Tree: 4

**Nr Apples:**	**Top Shape Class:**	**Symmetry:**	**Shape category:**
100	3	normal	rectangular

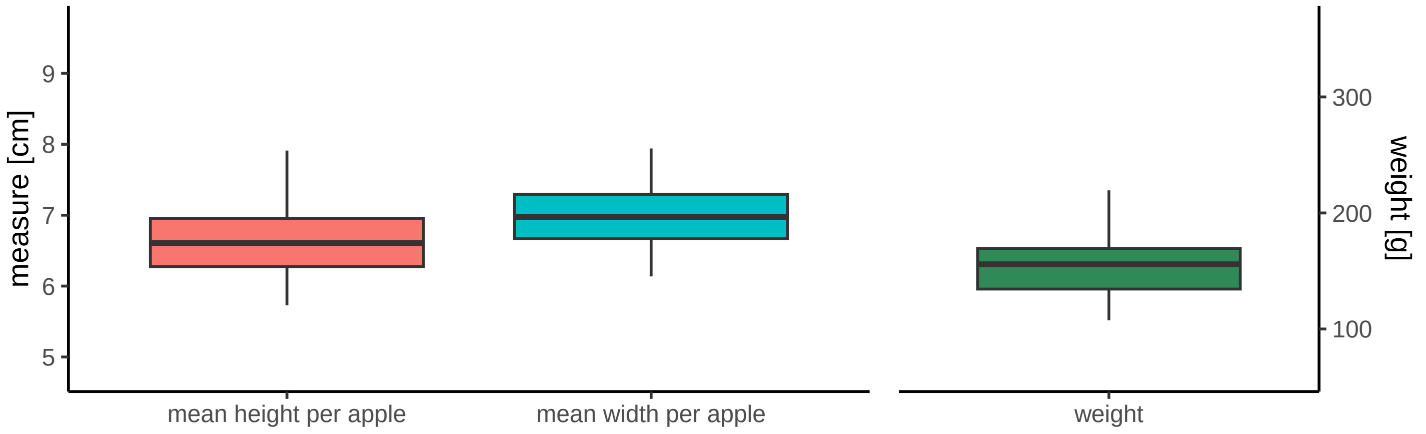

Top shapes

Average side shapes

Overall average side shape

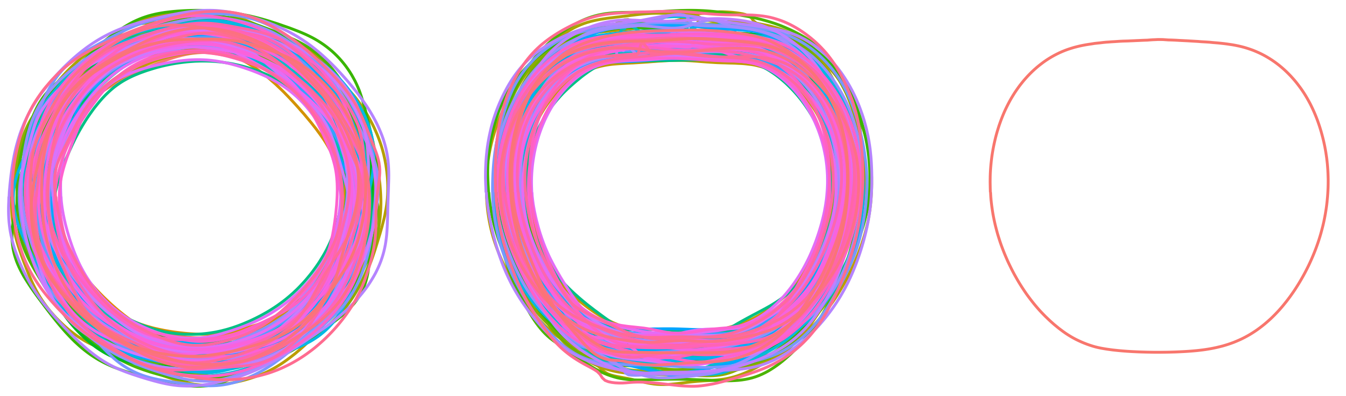

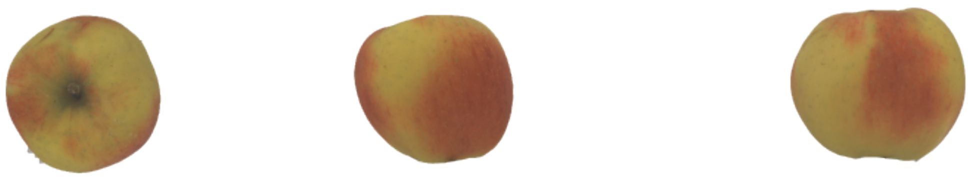

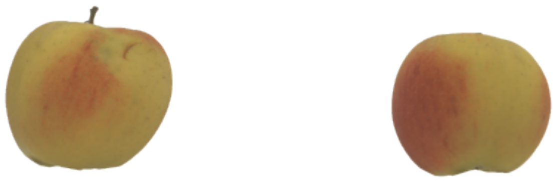

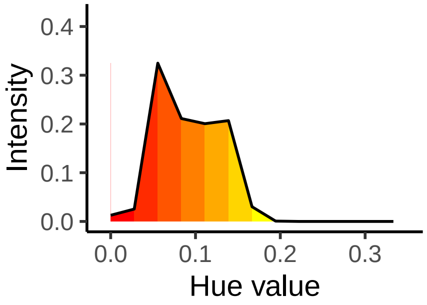

Location: Wädenswil
Year: 2021

Photos taken on 2021−12−17
between 15:00:52 and 15:16:41

Cripps Pink

Row: 5
Tree: 5

Nr Apples:	Top Shape Class:	Symmetry:	Shape category:
100	3	symmetric	rectangular

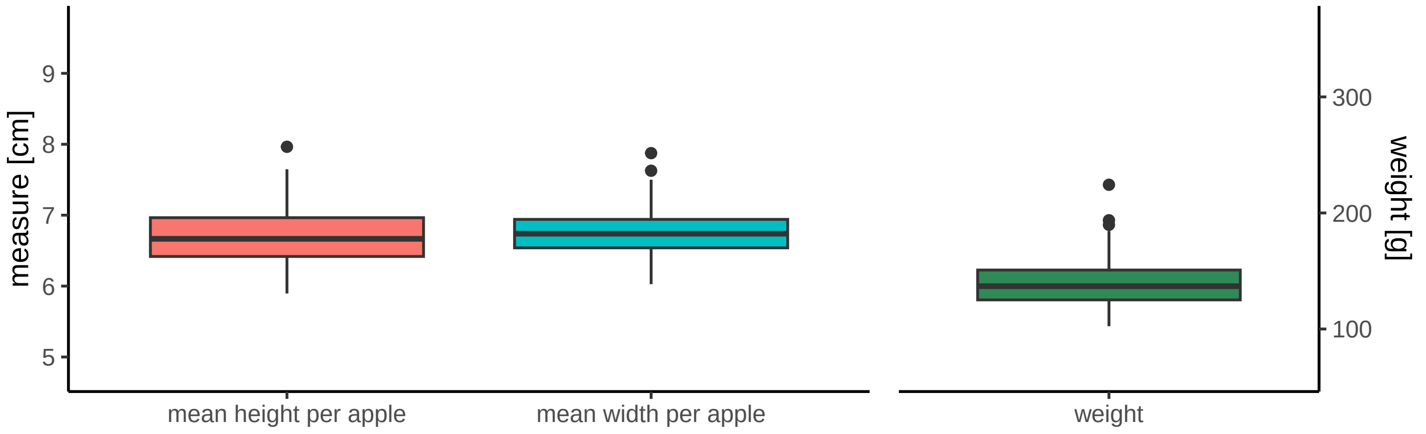

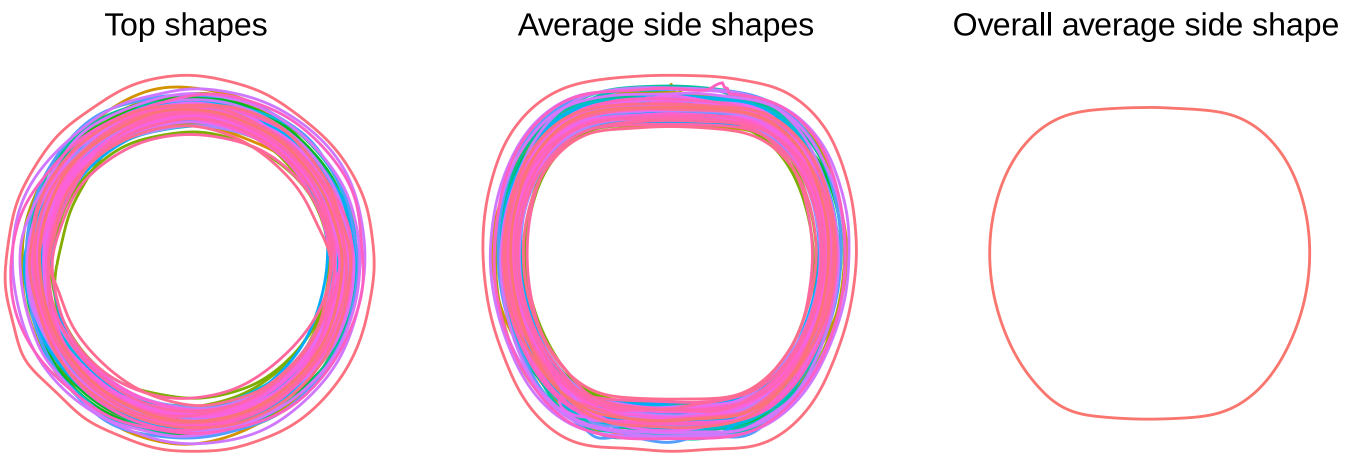

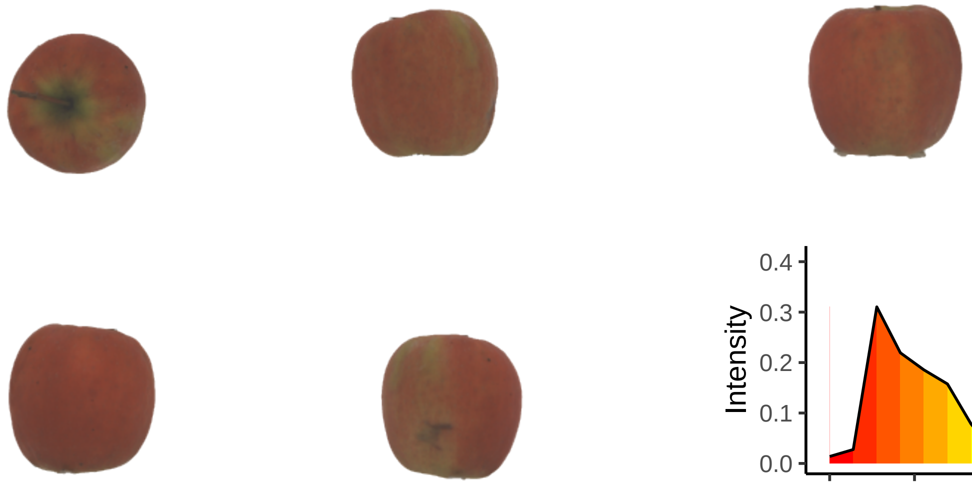

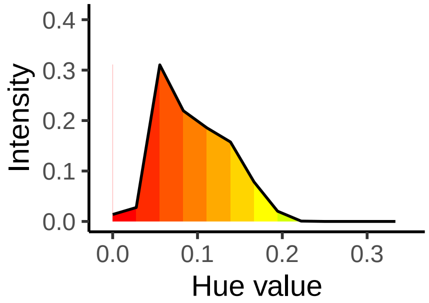

Location: Wädenswil
Year: 2021

Photos taken on 2021−12−17
between 13:37:47 and 14:00:57

Gala Galaxy

Row: 6
Tree: 6

Nr Apples:	Top Shape Class:	Symmetry:	Shape category:
100	3	normal	rectangular cone−shaped

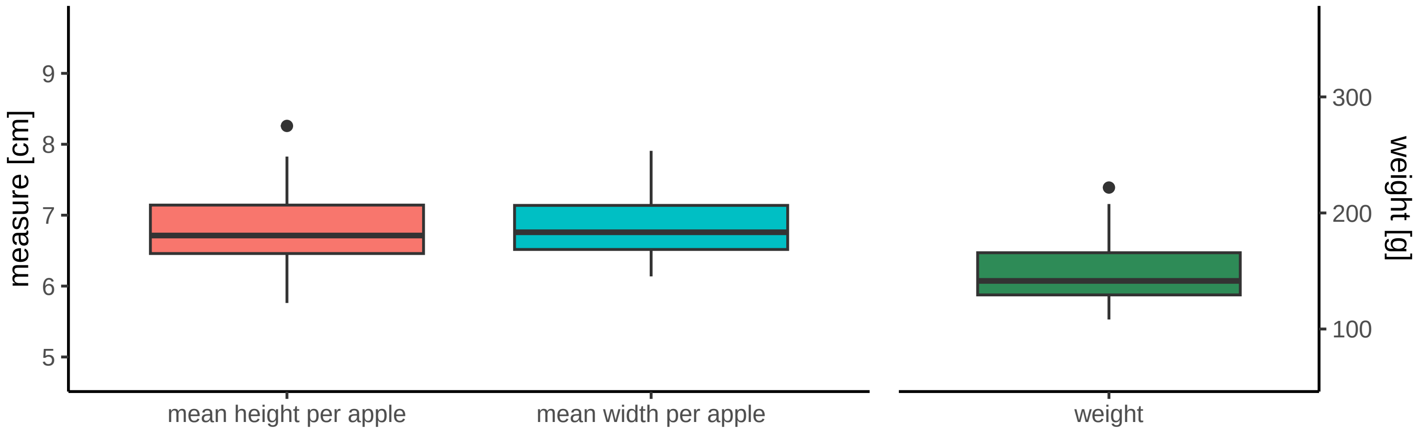

Top shapes

Average side shapes

Overall average side shape

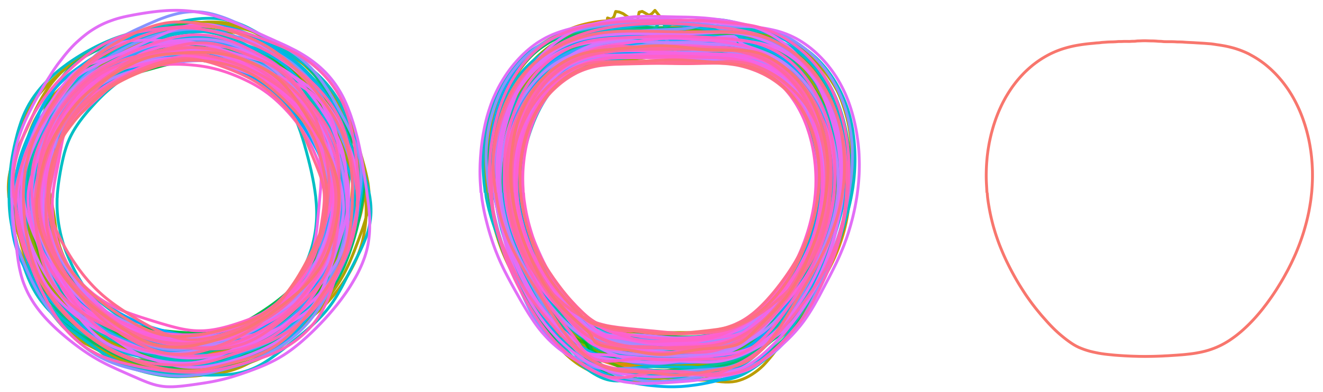

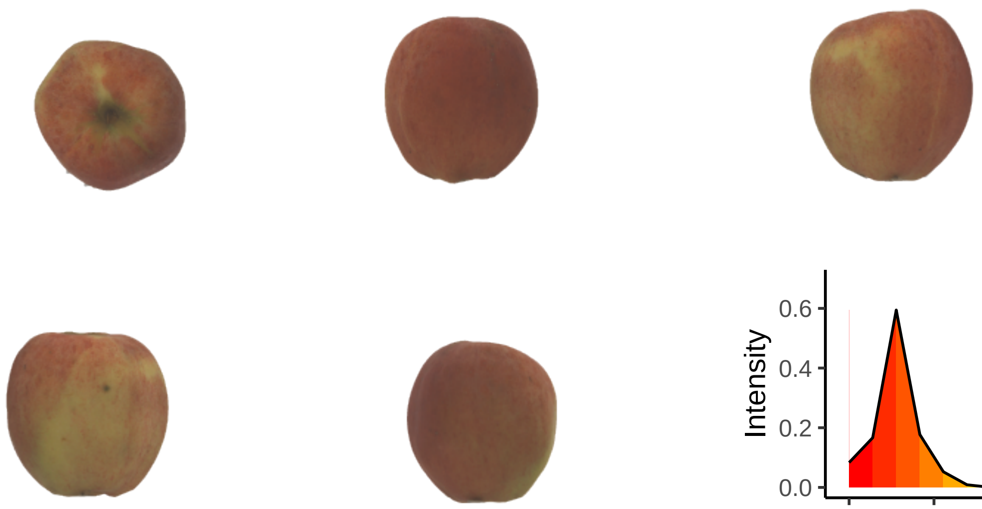

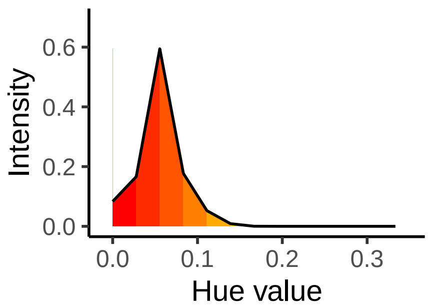

Location: Wädenswil
Year: 2021

Photos taken on 2021−12−17
between 14:20:23 and 14:37:10

Gala Schniga

Row: 7
Tree: 7

**Nr Apples:**	**Top Shape Class:**	**Symmetry:**	**Shape category:**
100	3	normal	rectangular cone−shaped

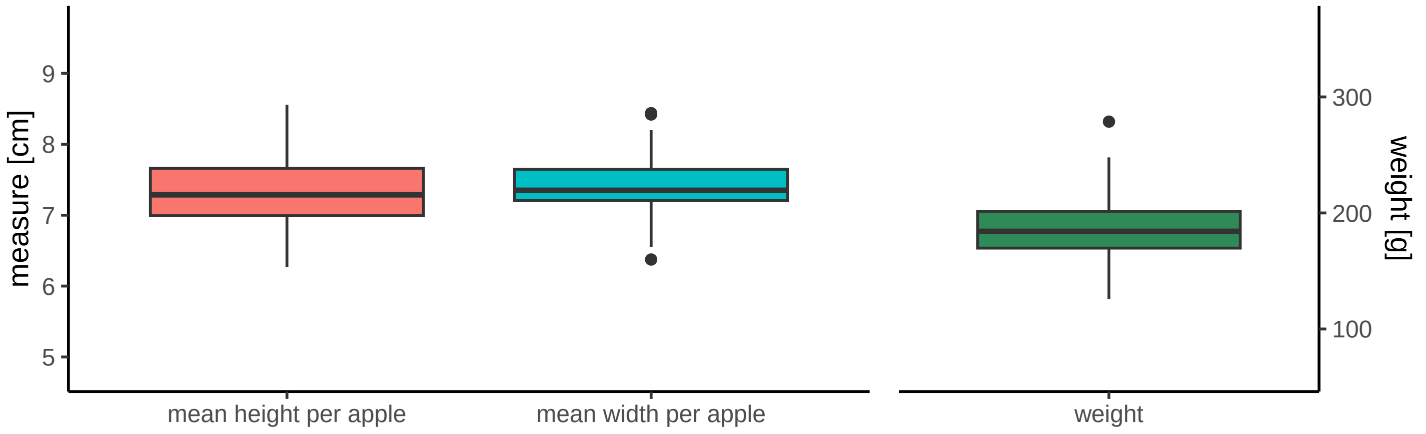

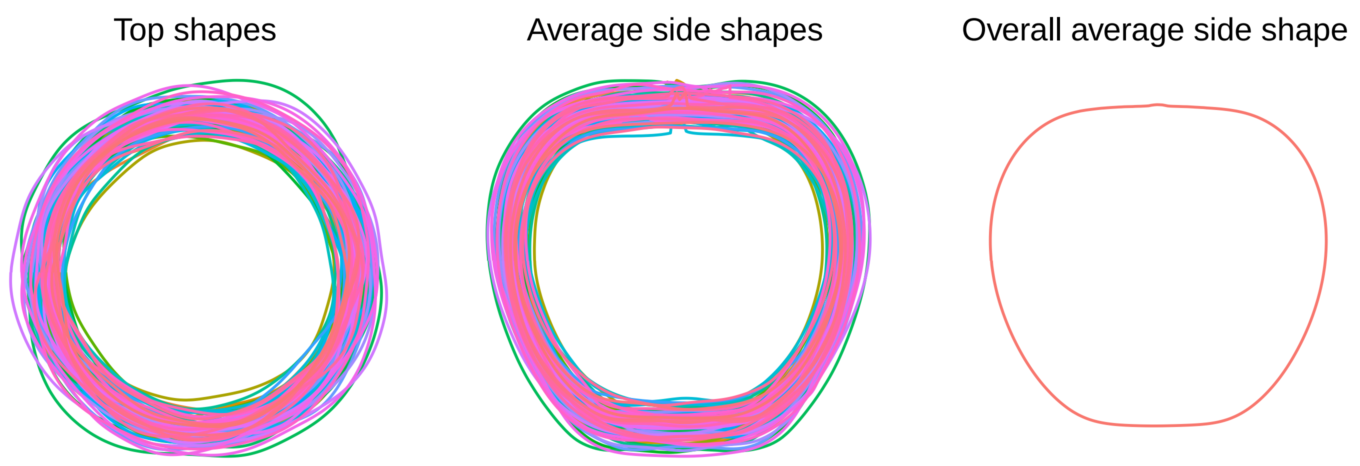

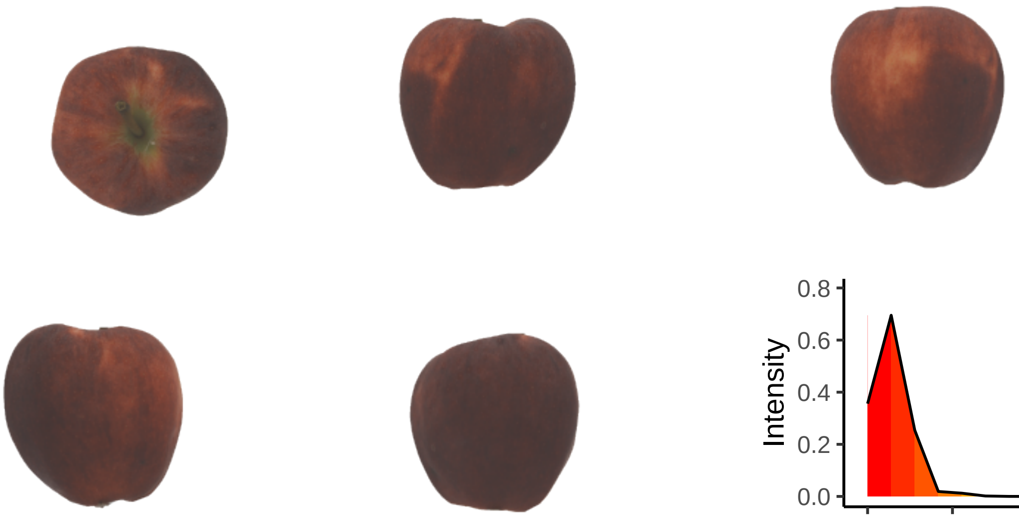

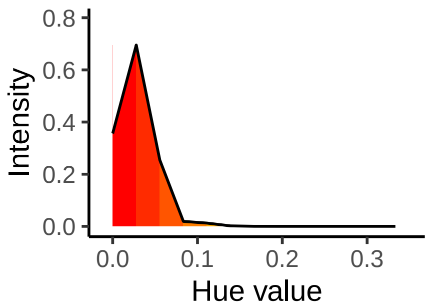

Location: Wädenswil
Year: 2021

Photos taken on 2021−12−17
between 10:47:54 and 11:06:04

Golden Reinders

Row: 15
Tree: 15

**Nr Apples:**	**Top Shape Class:**	**Symmetry:**	**Shape category:**
97	3	symmetric	rectangular cone−shaped

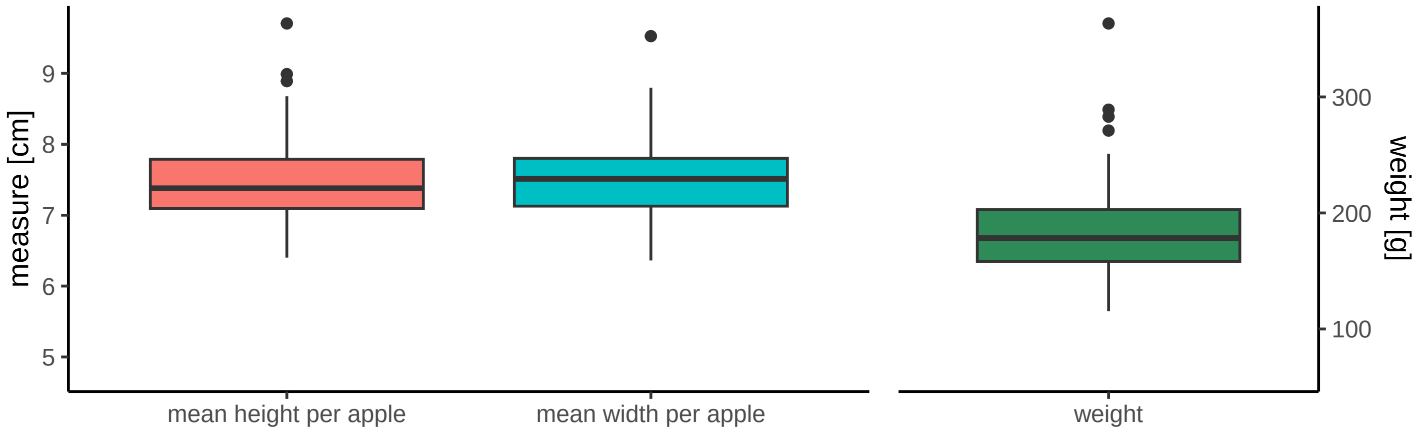

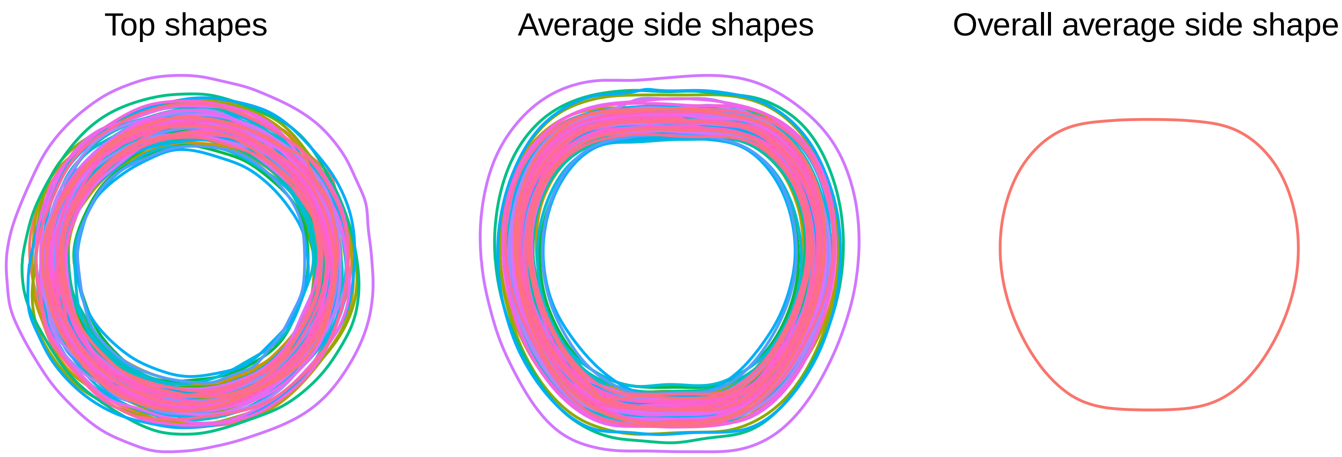

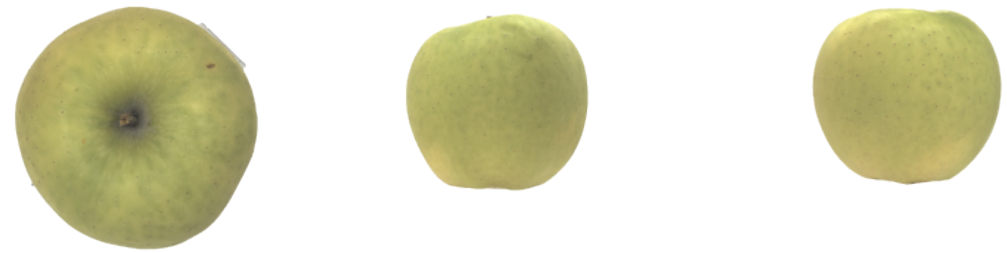

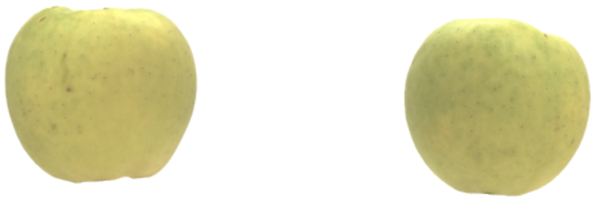

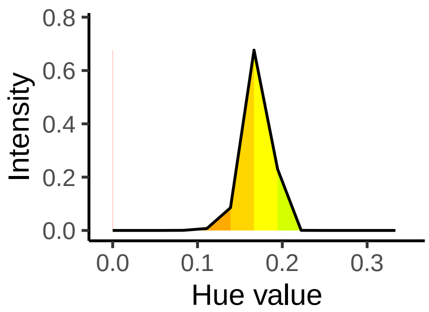

Location: Wädenswil
Year: 2021

Photos taken on 2021−01−06
between 16:20:40 and 16:39:20

Ladina

Row: 8

Tree: 8

**Nr Apples:**	**Top Shape Class:**	**Symmetry:**	**Shape category:**
100	3	normal	flattened conical

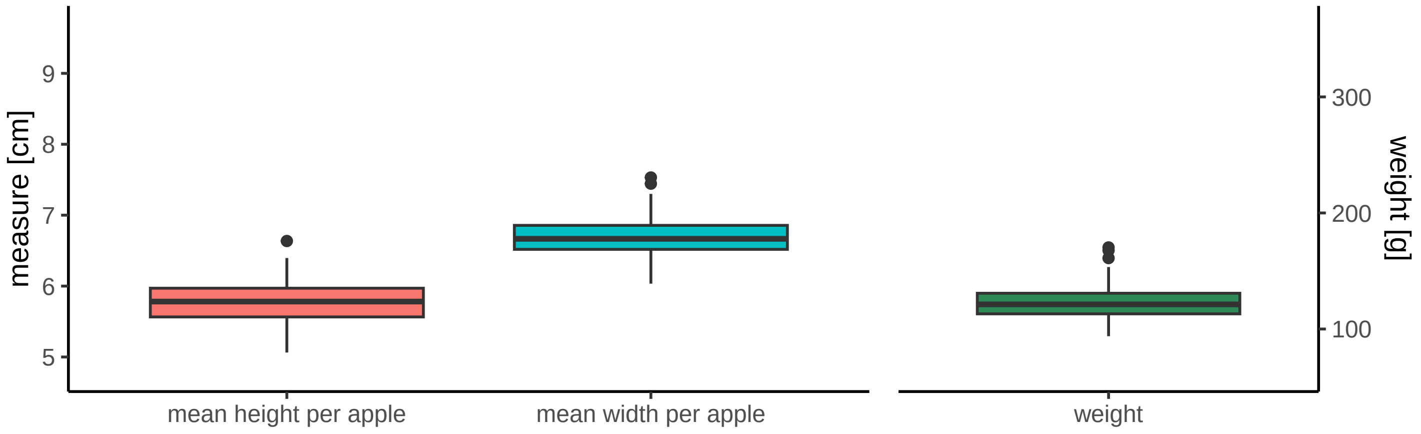

Top shapes

Average side shapes

Overall average side shape

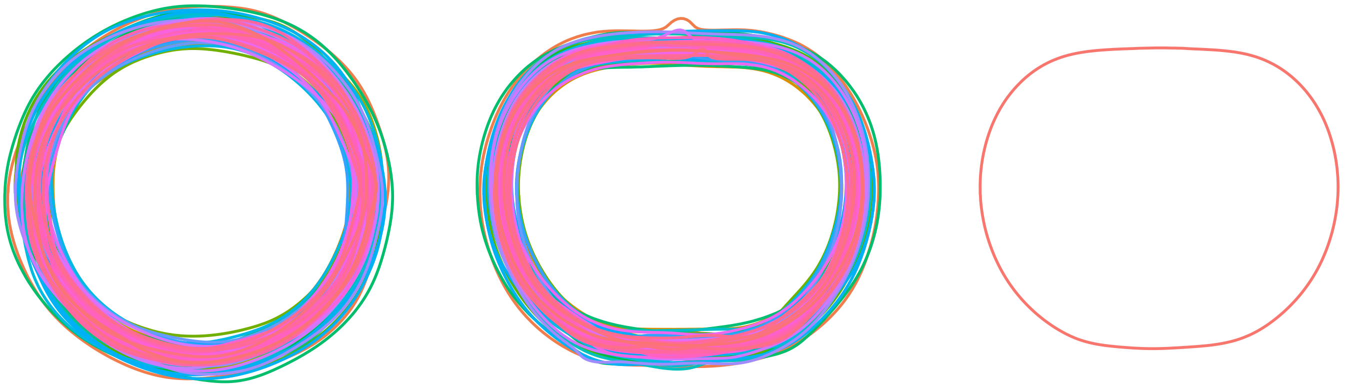

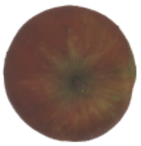

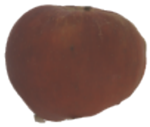

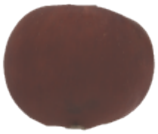

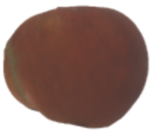

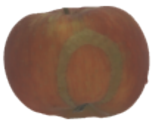

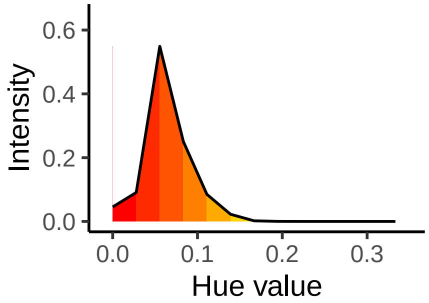

Location: Wädenswil
Year: 2021

Photos taken on 2021−12−17
between 09:03:02 and 09:19:26

Mariella

Row: 9
Tree: 9

Nr Apples:	Top Shape Class:	Symmetry:	Shape category:
100	3	normal	rectangular cone−shaped

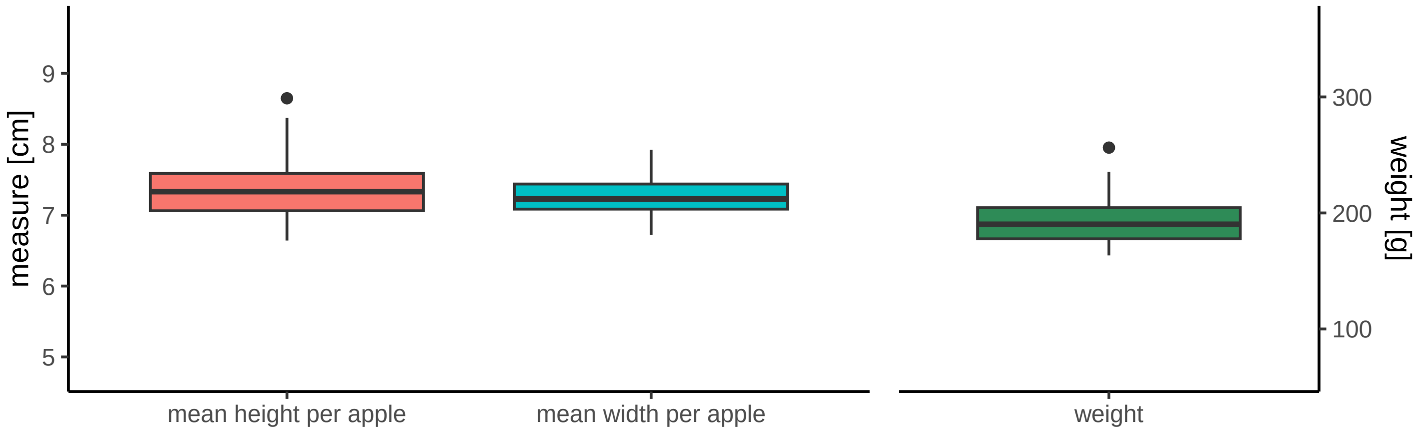

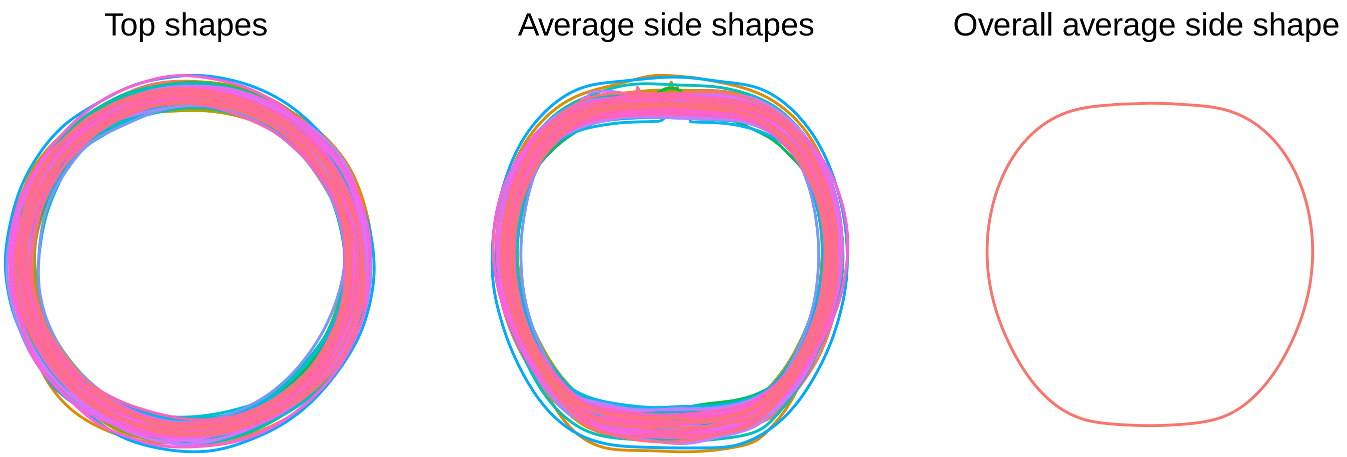

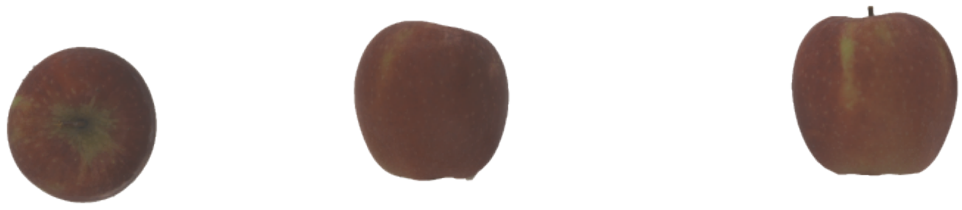

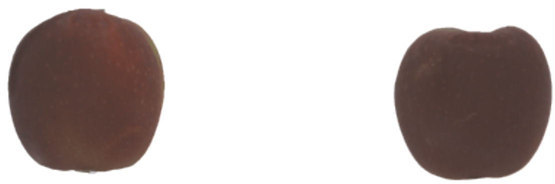

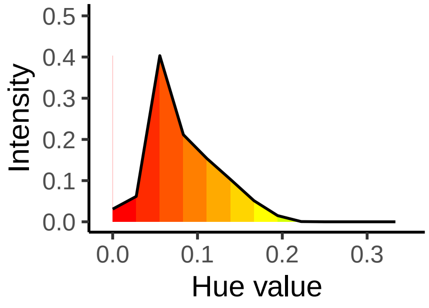

Location: Wädenswil
Year: 2021

Photos taken on 2021−12−17
between 14:01:48 and 14:15:57

Milwa

Row: 10
Tree: 10

**Nr Apples:**	**Top Shape Class:**	**Symmetry:**	**Shape category:**
100	3	normal	flattened conical

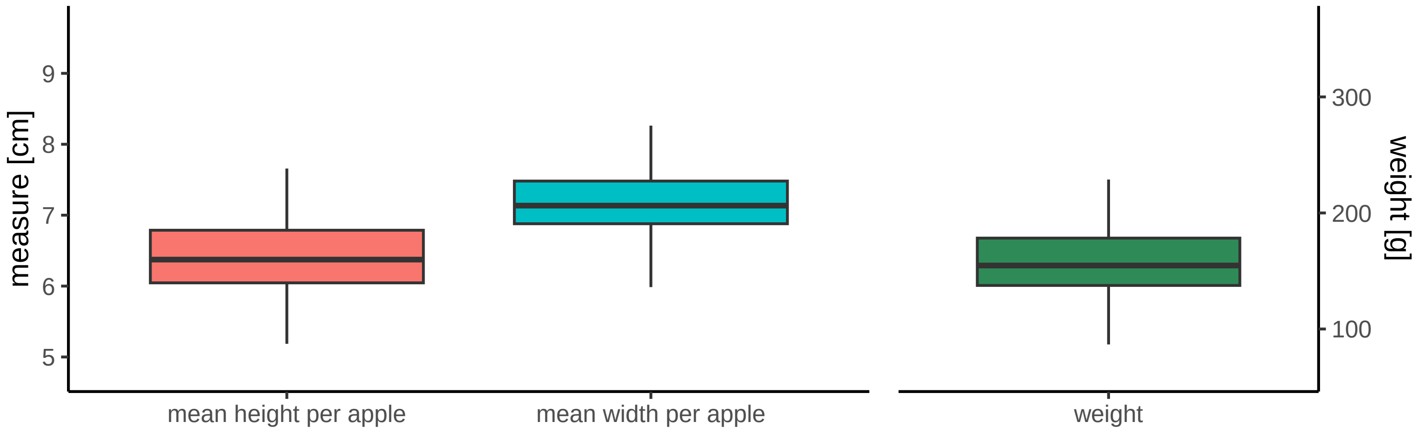

Top shapes

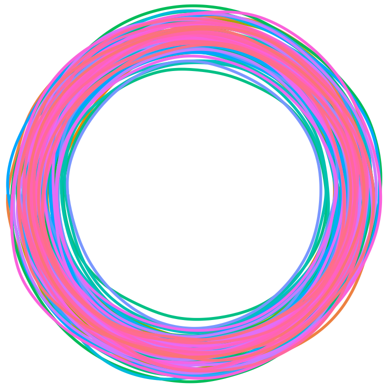

Average side shapes

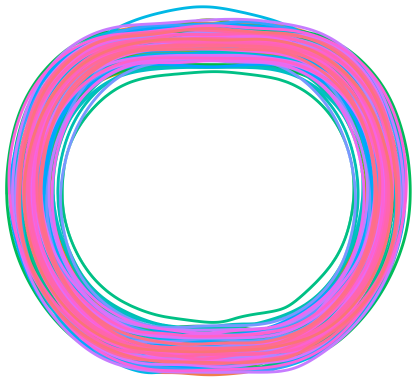

Overall average side shape

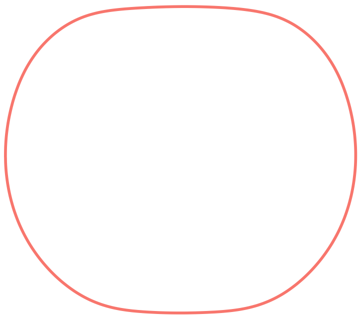

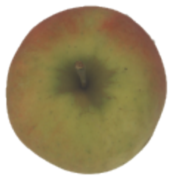

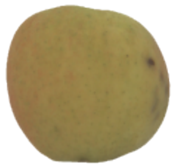

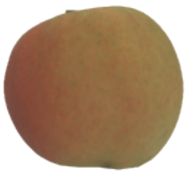

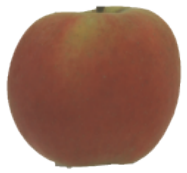

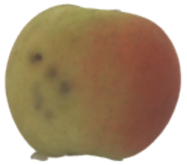

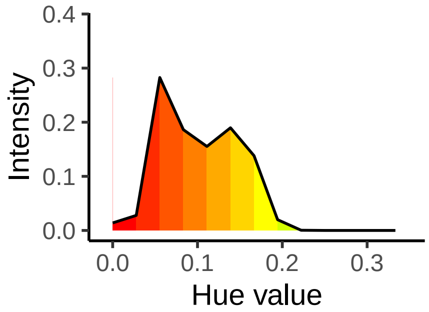

Location: Wädenswil
Year: 2021

Photos taken on 2021−12−17
between 09:20:31 and 09:37:21

PremA96

Row: 11
Tree: 11

Nr Apples:	Top Shape Class:	Symmetry:	Shape category:
100	3	normal	rectangular

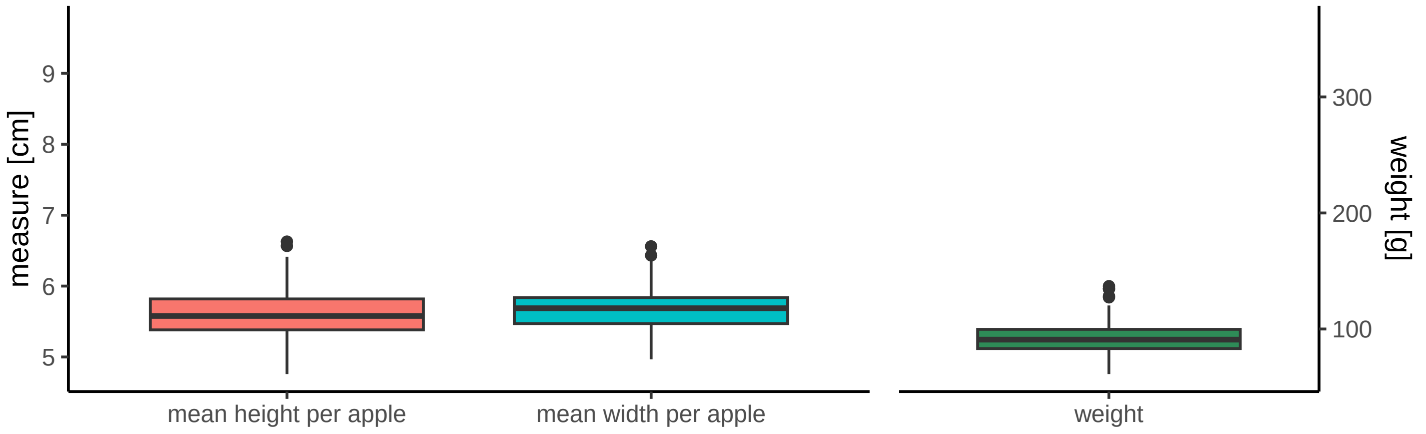

Top shapes

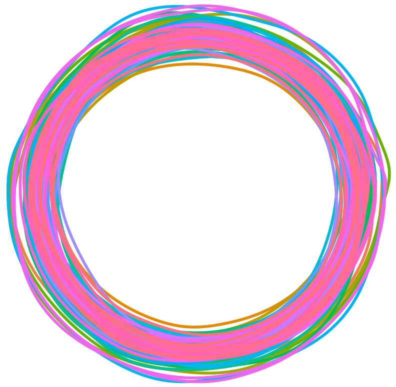

Average side shapes

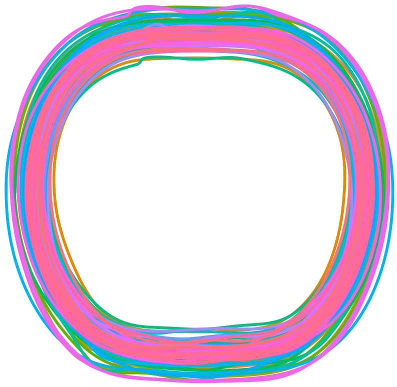

Overall average side shape

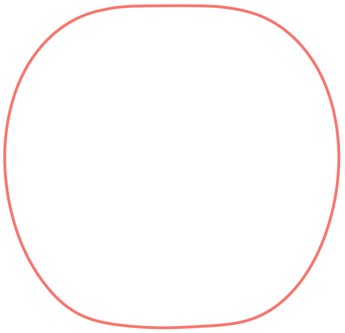

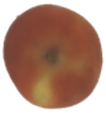

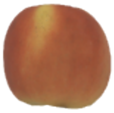

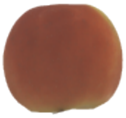

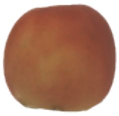

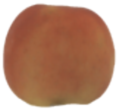

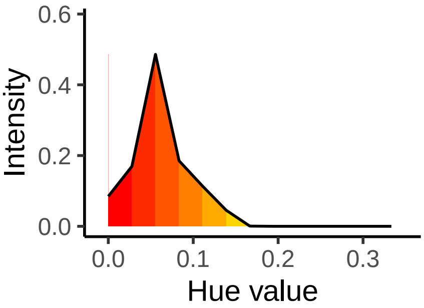

Location: Wädenswil
Year: 2021

Photos taken on 2021−12−17
between 08:46:44 and 09:00:24

Rustica

Row: 12
Tree: 12

**Nr Apples:**	**Top Shape Class:**	**Symmetry:**	**Shape category:**
100	3	symmetric	flattened conical

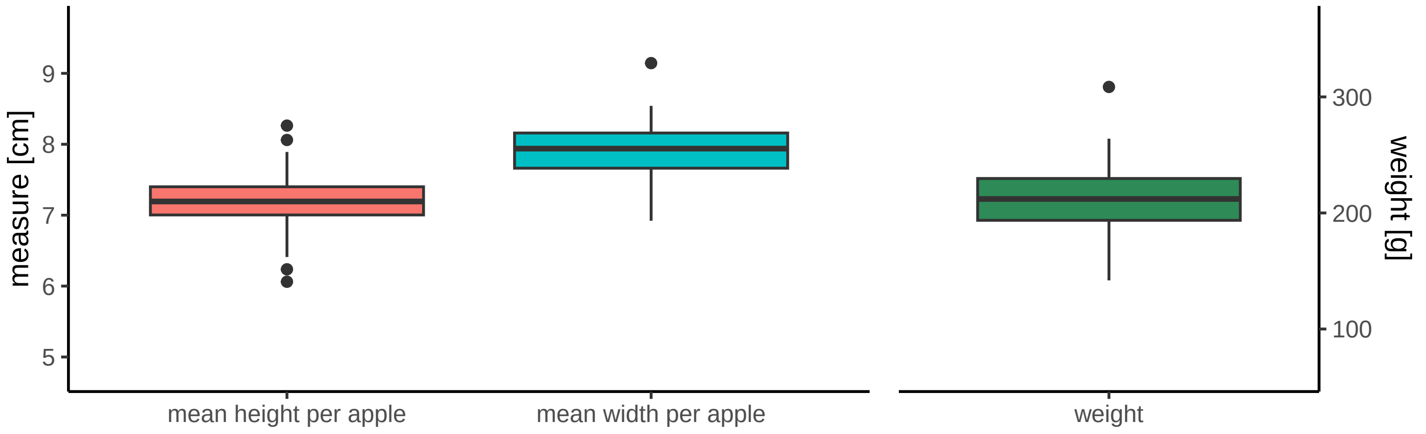

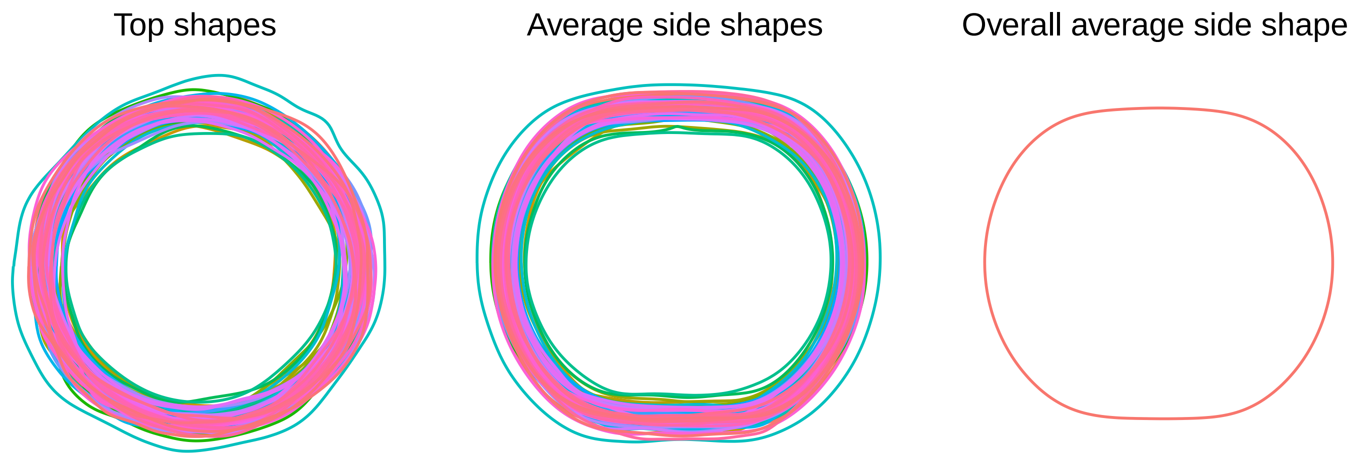

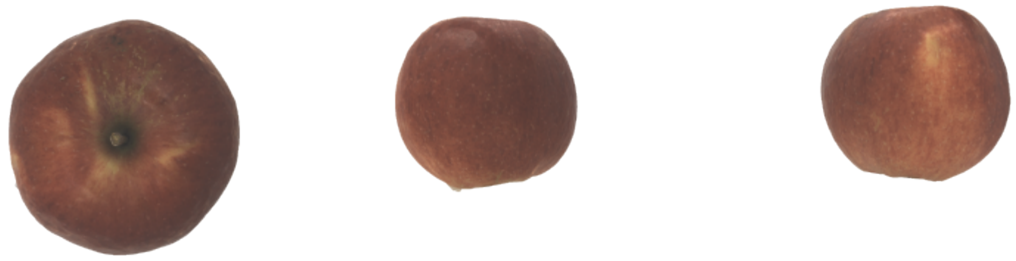

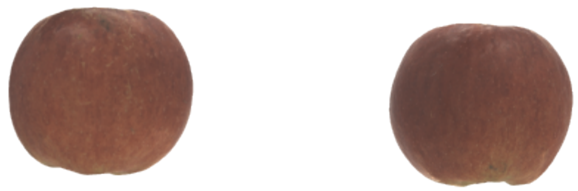

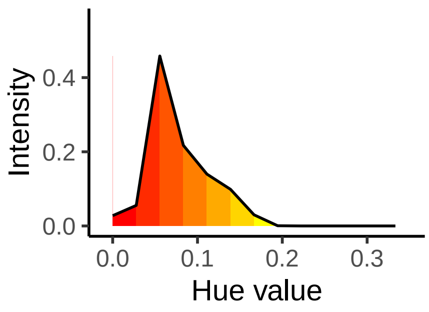

Location: Wädenswil
Year: 2021

Photos taken on 2021−01−06
between 15:27:15 and 15:46:46

SQ 159

Row: 13

Tree: 13

Nr Apples:	Top Shape Class:	Symmetry:	Shape category:
100	3	symmetric	conical

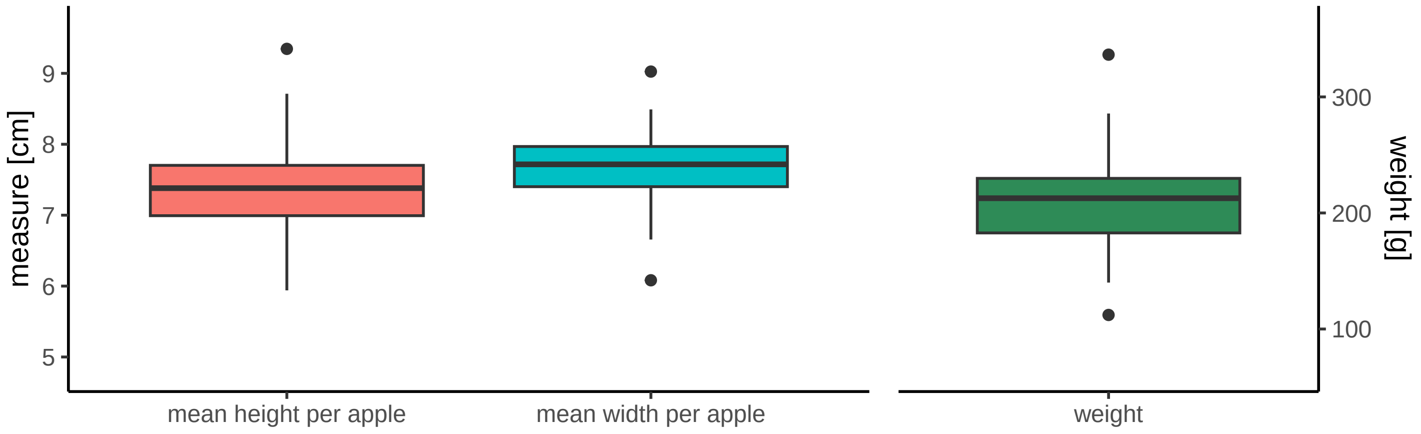

Top shapes

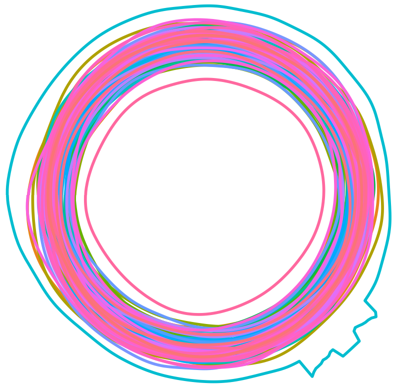

Average side shapes

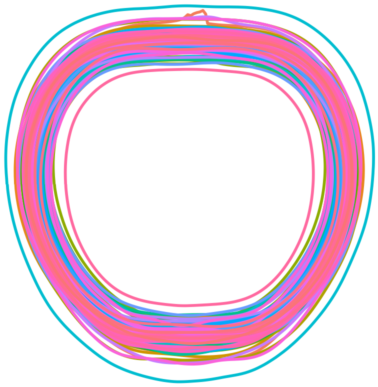

Overall average side shape

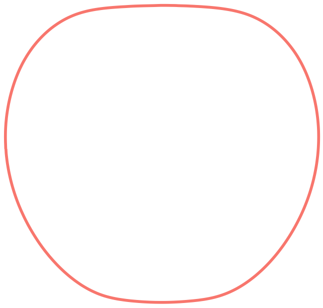

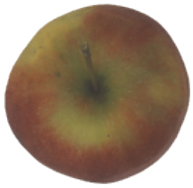

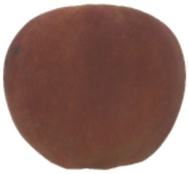

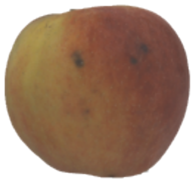

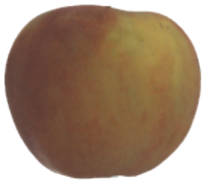

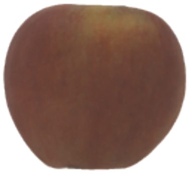

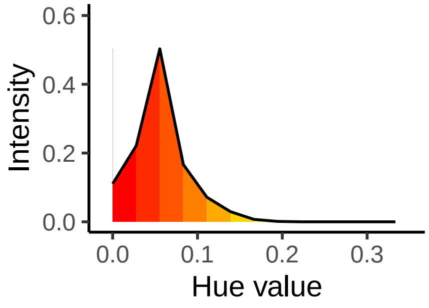

Location: Wädenswil
Year: 2021

Photos taken on 2021−12−17
between 10:27:26 and 10:42:36

Topaz

Row: 14
Tree: 14

**Nr Apples:**	**Top Shape Class:**	**Symmetry:**	**Shape category:**
100	3	normal	flattened conical

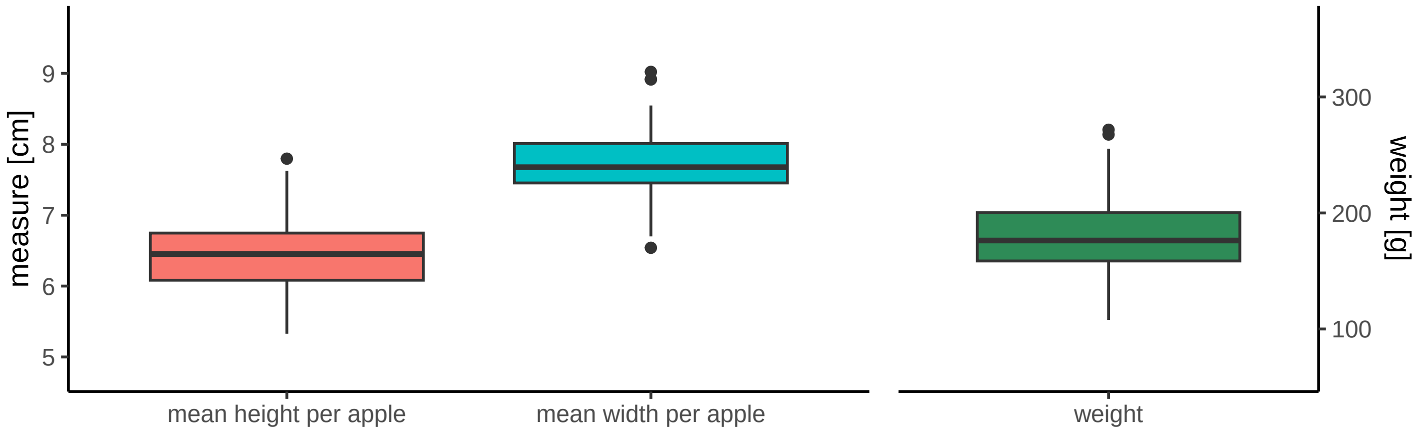

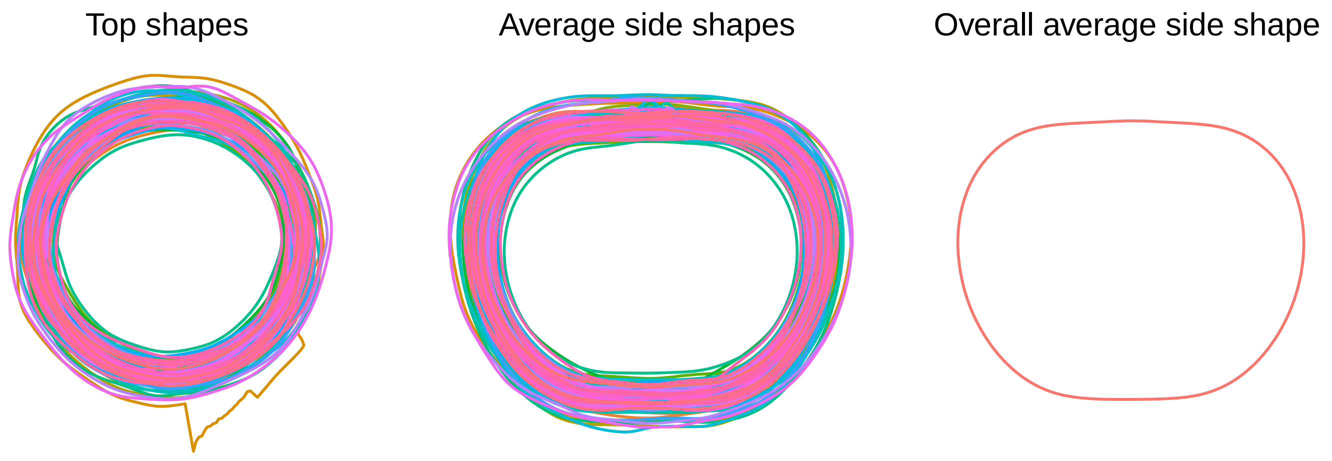

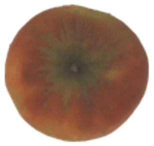

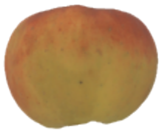

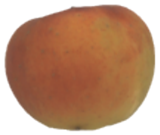

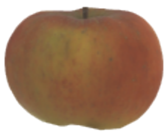

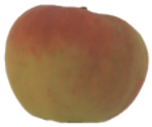

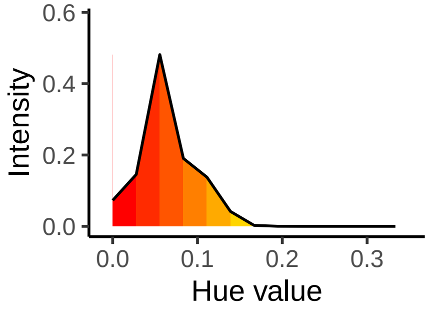

Location: Wädenswil
Year: 2021

Photos taken on 2021−12−17
between 14:38:10 and 14:57:39
