# Supplementary material for: FruitPhenoBox – a device for rapid and automated fruit phenotyping of small sample sizes
Source: Plant Methods. 2024 May 23;20:74. doi: 10.1186/s13007-024-01206-2 (PMC11112871; doi:10.1186/s13007-024-01206-2)
Supplement: Supplementary file 2 — Supplementary Material 2 [file 13007_2024_1206_MOESM2_ESM.docx]

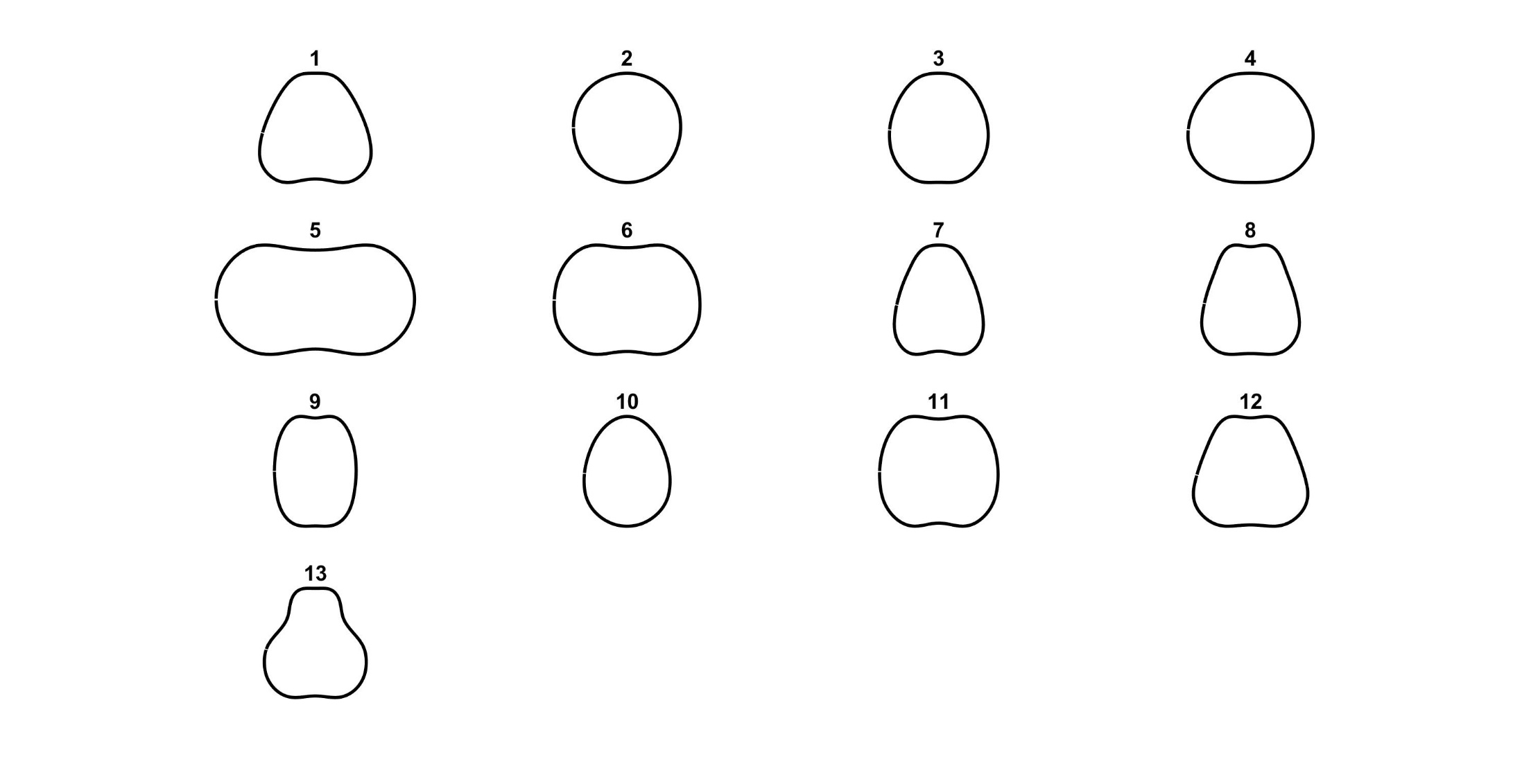


Figure S1: Images of the 13 reference hand-drawed shapes derived from the NAP-PGREL pomology description manual (Szalatnay D, Bauermeister R: **Obst-Deskriptoren NAP** ). Shape names are following. 1. Conical, 2. Spherical, 3. Spherico-conical, 4. Broad spherico-conical, 5. Flattened, 6. Flattened spherical, 7. Narrow conical, 8. Blunt conical, 9. Barrel-shaped, 10. Egg-shaped, 11. Rectangular, 12. Rectangular conical, 13. Bell-shaped. Reproduceed from.


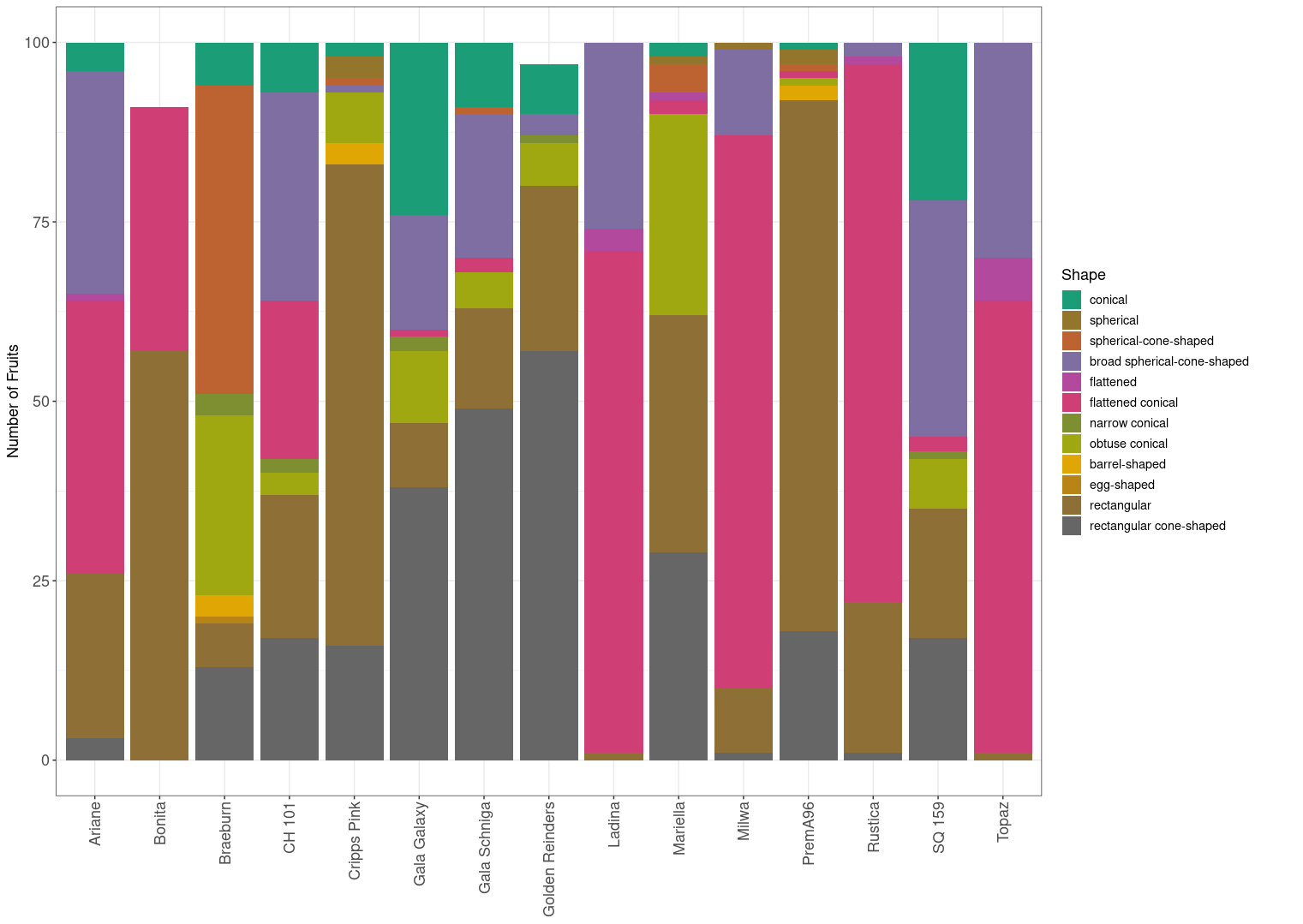


Figure S2: Cumulative bar representation of the number of fruits assigned to one of the six shape clusters. Bar colors indicate the shape cluster to which each single average fruit side contour was assigned to.


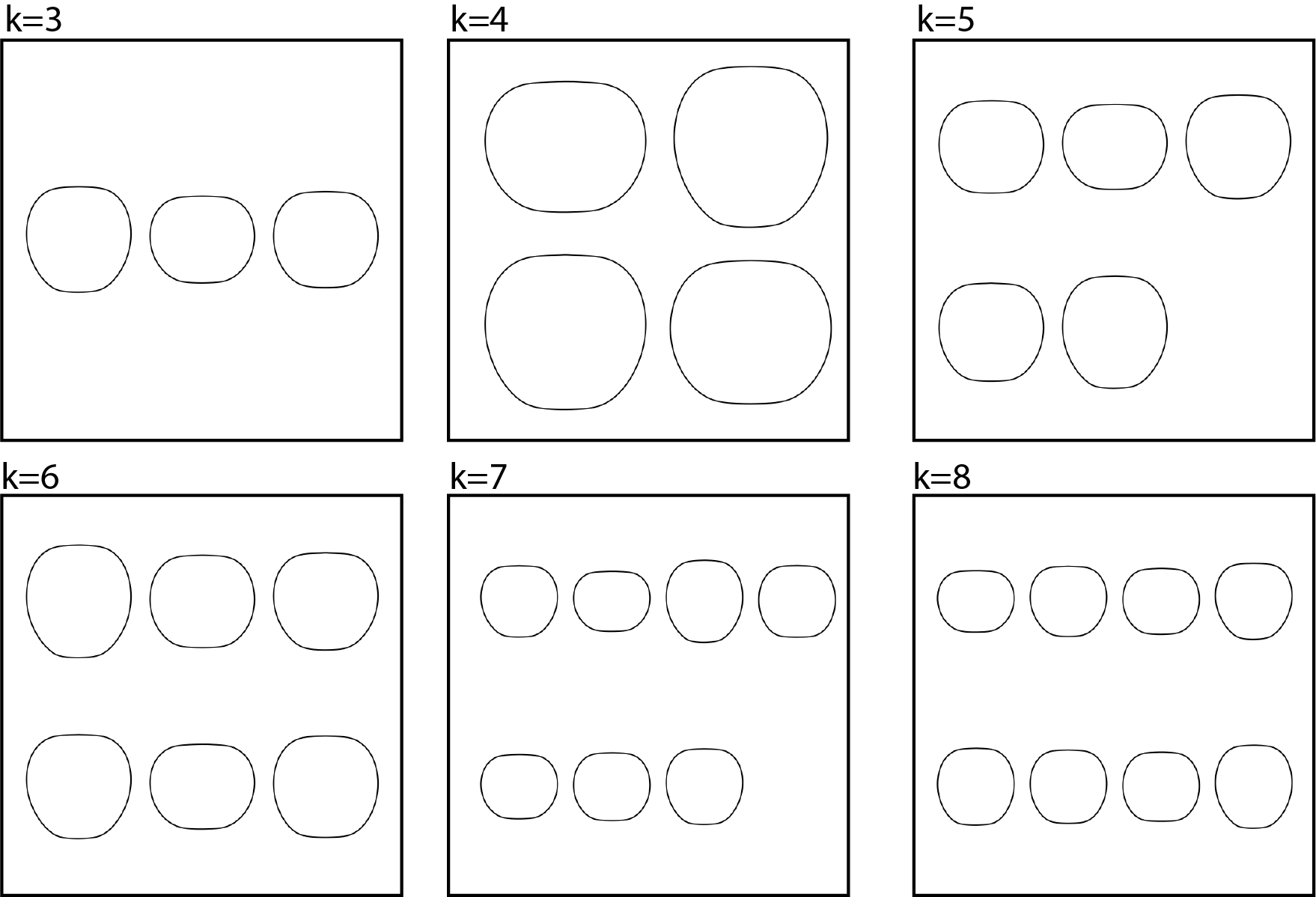


Figure S3: Representation of fruit side contours clusters (not in scale) according to k-mean clustering approach generated using approximately 100 fruit from 15 different cultivars each. k= number of clusters in which fruit side contours are partitioned.


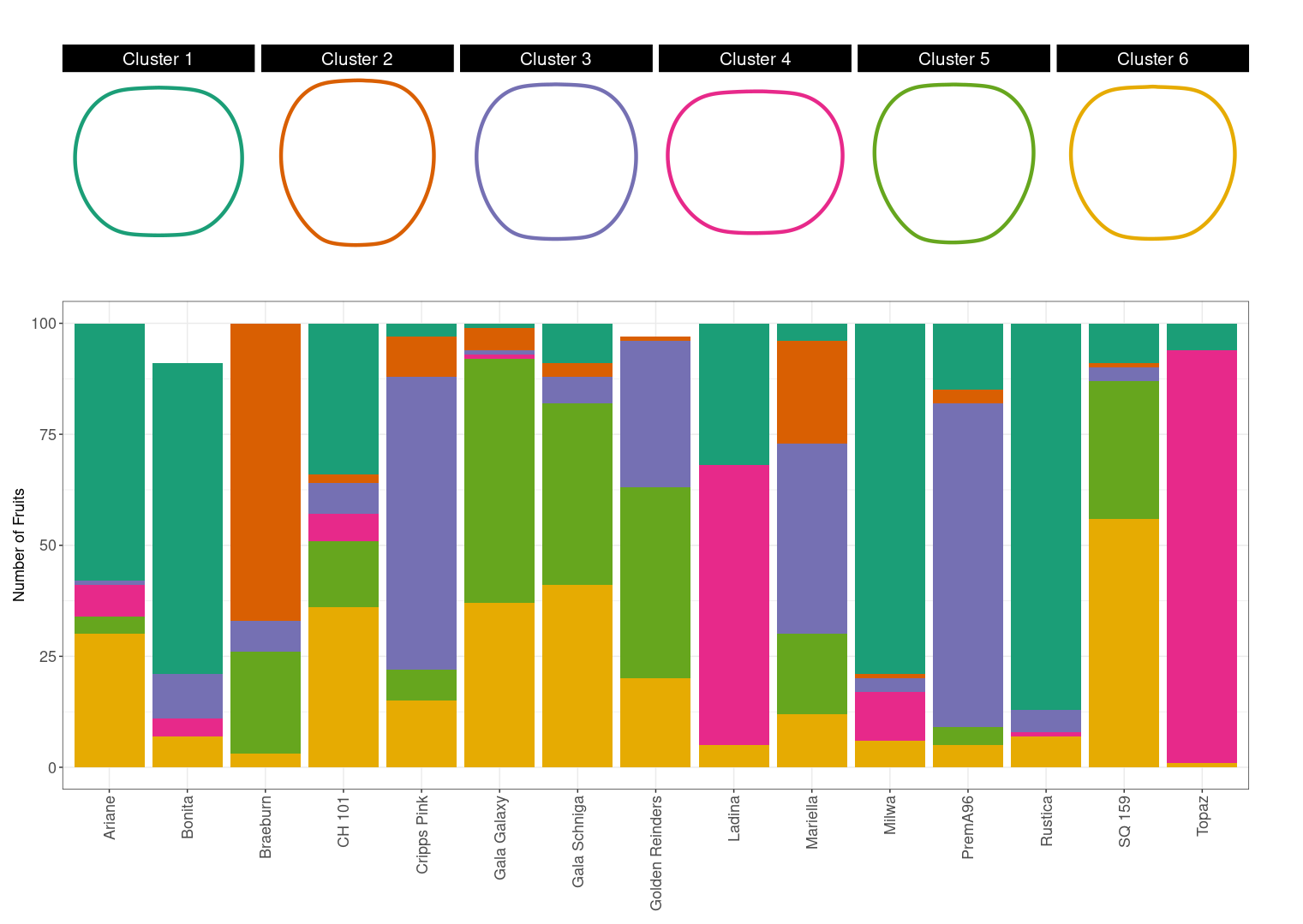


Figure S4: Cumulative bar representation of the number of fruits assigned to one of the six shape clusters. Bar colors indicate the shape cluster to which each single average fruit side contour was assigned to.

.
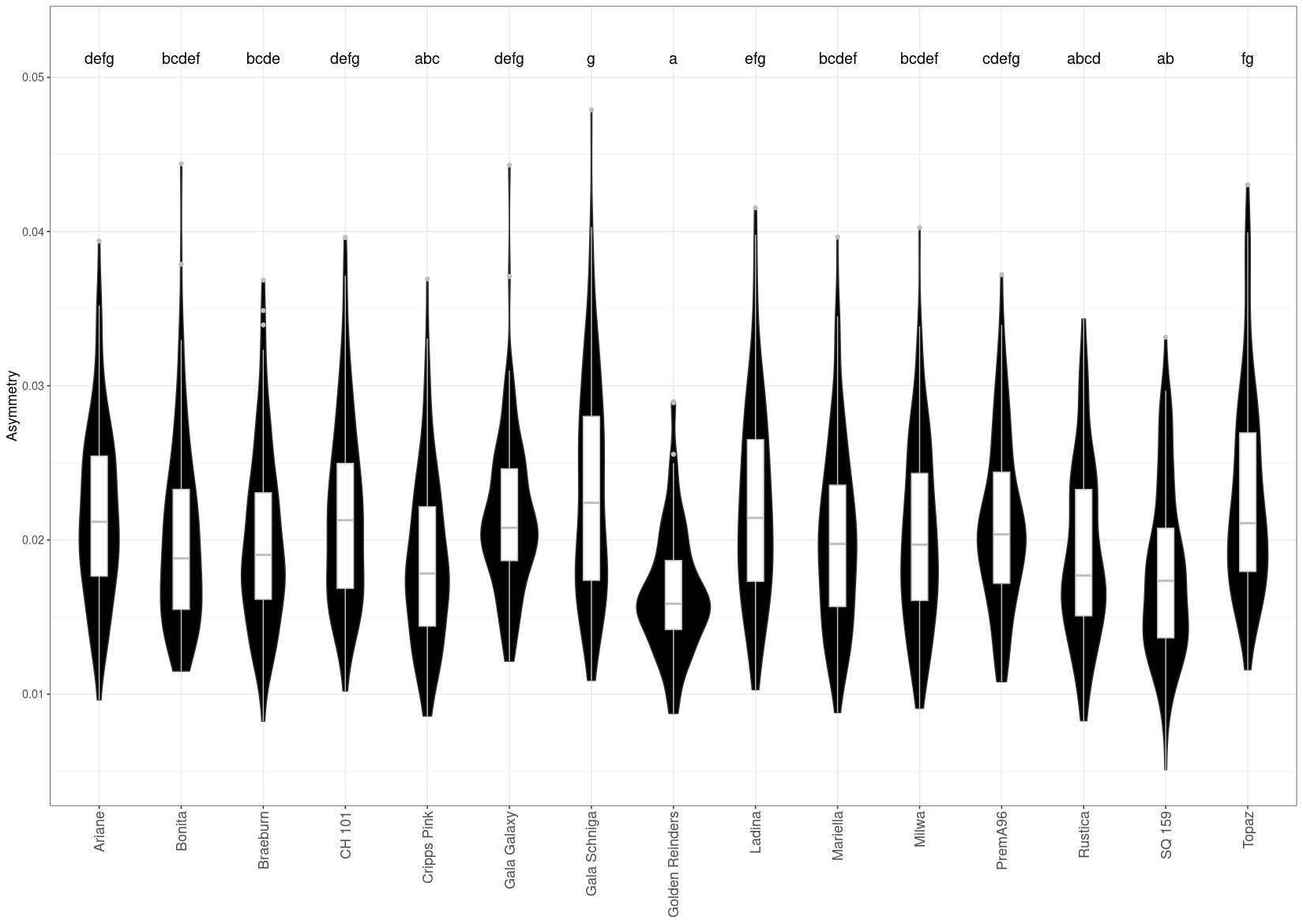


Figure S5: Violin plot overlayed with boxplot representation of the distribution of the fruit asymmetry for each of the 15 apple cultivars. Letters above the boxes indicate significance groups (*p* < 0.05) according to a post hoc Tukey test.


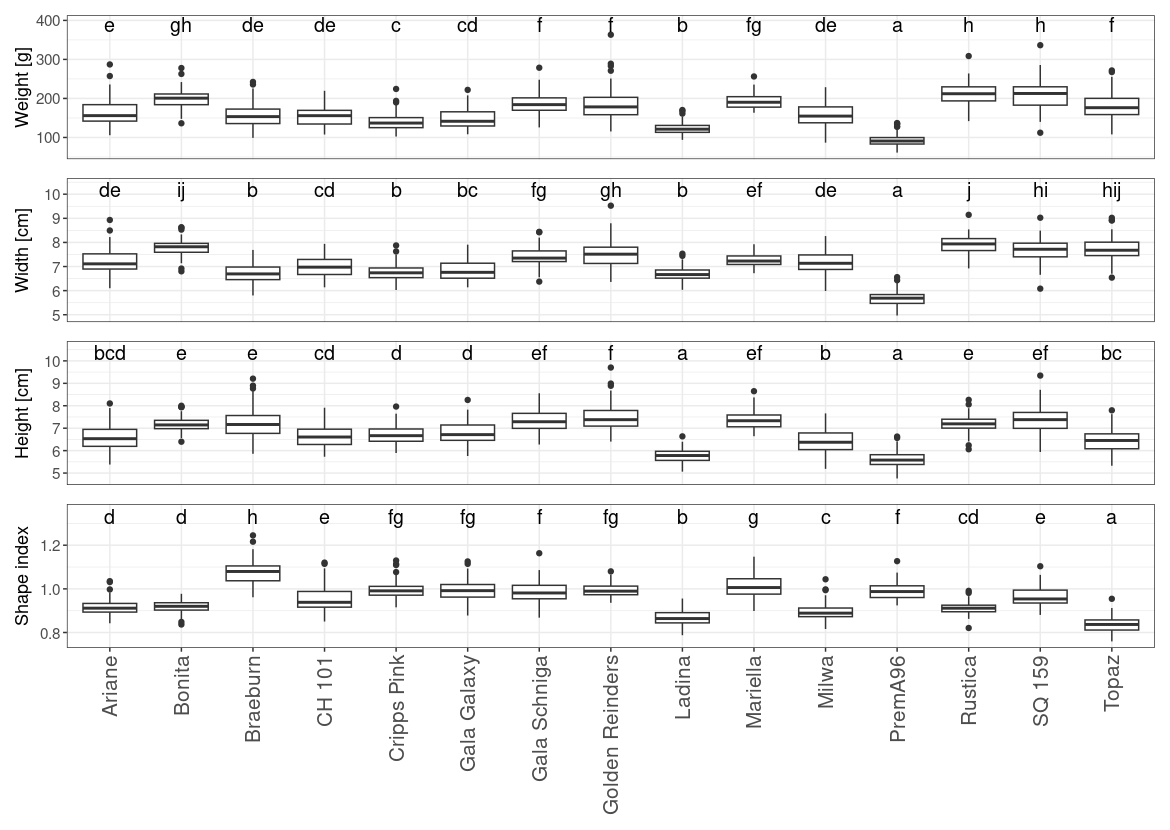


Figure S6: Boxplot representation of the distribution of weight, width, height, and shape index (ratio width/height) for each of the 15 apple cultivars. Letters above the boxes indicate significance groups (*p* < 0.05) according to a post hoc Tukey test.


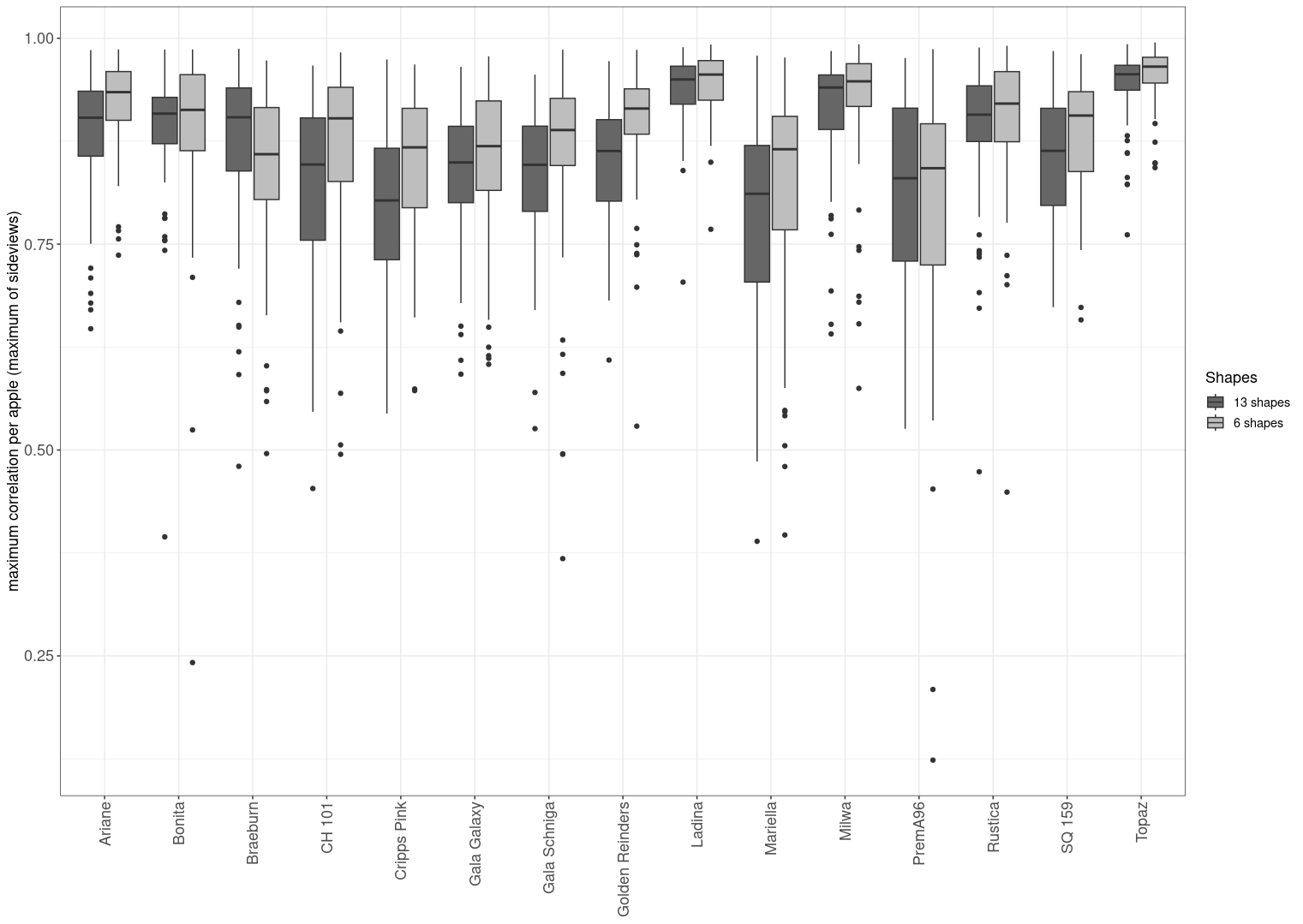


Figure S7: Boxplot representation of the maximum correlation of the side views of each fruit of a cultivar compared to the 13 reference shape descriptors shown in Figure S1 or to the six shape clusters derived from k-mean clustering (Figure S4) generated approximately from 1500 average fruit side views generated in this work. Dots represent outliers.

id Position Cultivar_label Row Genotype

Ariane 1 Ariane 1 1

Bonita 2 Bonita 2 2

Braeburn 3 Braeburn 3 3

CH 101 4 CH 101 4 4

Cripps Pink 5 Cripps Pink 5 5

Gala Galaxy 6 Gala Galaxy 6 6

Gala Schniga 7 Gala Schniga 7 7

Golden Reinders 15 Golden Reinders 15 15

Ladina 8 Ladina 8 8

Mariella 9 Mariella 9 9

Milwa 10 Milwa 10 10

PremA96 11 PremA96 11 11

Rustica 12 Rustica 12 12

SQ 159 13 SQ 159 13 13

Topaz 14 Topaz 14 14

Table S1: Example of tab-separated text file with the plan of the experimental site including tree name/ID, cultivar name, row and tree position required as an additional input file for generating a factsheet.
